# Efficacy of Cold Atmospheric Plasma vs. Chemotherapy in Triple-Negative Breast Cancer: A Systematic Review

**DOI:** 10.3390/ijms25063254

**Published:** 2024-03-13

**Authors:** Catarina Almeida-Ferreira, Carlos Miguel Marto, Chrislaura Carmo, Joana Almeida-Ferreira, Cristina Frutuoso, Maria João Carvalho, Maria Filomena Botelho, Mafalda Laranjo

**Affiliations:** 1 Institute for Clinical and Biomedical Research (iCBR), Area of Environment Genetics and Oncobiology (CIMAGO), Faculty of Medicine, University of Coimbra, 3000-548 Coimbra, Portugal; catarinalmeidaferreira@gmail.com (C.A.-F.); cmiguel.marto@uc.pt (C.M.M.); cribego@gmail.com (C.C.); cmffrutuoso@gmail.com (C.F.); mariajoaosflcarvalho@gmail.com (M.J.C.); mfbotelho@fmed.uc.pt (M.F.B.); 2 Institute of Biophysics, Faculty of Medicine, University of Coimbra, 3000-548 Coimbra, Portugal; 3 Center for Innovative Biomedicine and Biotechnology (CIBB), 3000-548 Coimbra, Portugal; 4 Faculty of Pharmacy, University of Coimbra, 3000-548 Coimbra, Portugal; 5 Clinical Academic Center of Coimbra (CACC), University of Coimbra, 3000-354 Coimbra, Portugal; 6 Institute of Integrated Clinical Practice, Faculty of Medicine, University of Coimbra, 3000-354 Coimbra, Portugal; 7 Laboratory for Evidence-Based Sciences and Precision Dentistry, Faculty of Medicine, University of Coimbra, 3000-075 Coimbra, Portugal; 8 Institute of Experimental Pathology, Faculty of Medicine, University of Coimbra, 3000-354 Coimbra, Portugal; 9 Coimbra Chemistry Center (CQC), Department of Chemistry, Faculty of Sciences and Technology, University of Coimbra, 3004-535 Coimbra, Portugal; 10 University Hospital Center of Central Lisbon, 1150-199 Lisbon, Portugal; joanalmeidaferreira@gmail.com; 11 Gynecology Service, Coimbra Hospital and University Centre, Coimbra Health Local Unit, 3004-561 Coimbra, Portugal; 12 Universitary Clinic of Gynecology, Faculty of Medicine, University of Coimbra, 3000-548 Coimbra, Portugal

**Keywords:** cold atmospheric plasma, chemotherapy drugs, systematic review, cell viability, triple-negative breast neoplasms, animal models

## Abstract

Breast cancer is a growing disease, with a high worldwide incidence and mortality rate among women. Among the various types, the treatment of triple-negative breast cancer (TNBC) remains a challenge. Considering the recent advances in cold atmospheric plasma (CAP) cancer research, our goal was to evaluate efficacy data from studies based on chemotherapy and CAP in TNBC cell lines and animal models. A search of the literature was carried out in the PubMed, Web of Science, Cochrane Library, and Embase databases. Of the 10,999 studies, there were fifty-four in vitro studies, three in vivo studies, and two in vitro and in vivo studies included. MDA-MB-231 cells were the most used. MTT, MTS, SRB, annexin-V/propidium iodide, trypan blue, and clonogenic assay were performed to assess efficacy in vitro, increasing the reliability and comprehensiveness of the data. There was found to be a decrease in cell proliferation after both chemotherapy and CAP; however, different protocol settings, including an extensive range of drug doses and CAP exposure times, were reported. For both therapies, a considerable reduction in tumor volume was observed in vivo compared with that of the untreated group. The treatment of TNBC cell lines with CAP proved successful, with apoptosis emerging as the predominant type of cellular death. This systematic review presents a comprehensive overview of the treatment landscape in chemotherapy and CAP regarding their efficacy in TNBC cell lines.

## 1. Introduction

Cancer is a growing disease worldwide. In 2020, breast cancer (BC) had highest incidence among cancers in women and was responsible for the largest number of deaths across all age groups [1]. The expressions of estrogen receptor-, progesterone receptor-, and human epidermal growth factor receptor-related protein are primary determinants of BC biology. This profile, which is associated with various high-throughput techniques, is used for BC stratification, prognosis, and treatment [2]. Triple-negative breast cancer (TNBC) represents approximately 15–20% of all breast cancer molecular subtypes [3]. It is characterized by the absence of these three types of receptors [4] and tends to have a worse prognosis [5]. Accurate molecular classification of TNBC is crucial for risk stratification [6,7]. Six TNBC molecular subtypes have been proposed, each one with its own features and responses to standard treatment: two different basal-like types (basal-like 1 and basal-like 2), immunomodulatory, mesenchymal, mesenchymal stem-like, and luminal androgen receptors [8,9]. These updates have allowed for personalized treatment with enhanced specificity. Currently, TNBC therapy responds to conventional chemotherapy and monoclonal antibodies, for example, pembrolizumab and avelumab, in the presence of specific biological markers such as programmed death-ligand 1 [10,11]. According to the European Society for Medical Oncology (ESMO) and American Society for Clinical Oncology (ASCO) guidelines for the treatment of TNBC, (neo)adjuvant chemotherapy drugs are used in almost all cases [11,12]. There are different regimens in use, including doxorubicin or epirubicin in combination with cyclophosphamide and paclitaxel or docetaxel in combination with carboplatin. If residual disease or the presence of the *BRCA* gene mutations is positive, capecitabine or olaparib is also included as an option [11,12]. Unfortunately, TNBC treatment continues to be a challenge, and new approaches are still needed. Some patients present insufficient response, others develop resistance, and treatments are frequently associated with adverse effects [13]. 

The plasma state, also known as the fourth state of matter, has enough energy to ionize a significant amount of positive and negative particles [14]. There are two types of plasma, namely thermal and non-thermal; the latter is frequently referred to as “cold atmospheric plasma” (CAP). CAP is characterized as non-thermal because the heavy particles are at room temperature. Several methods for producing it have been described [15]. CAP has recently been studied as a potential cancer treatment, with evidence obtained in several malign neoplasms both in vitro and in vivo [16,17,18,19,20]. Some authors have demonstrated CAP selectivity to tumoral cells compared to non-malignant counterparts, highlighting its potential in cancer treatment [21,22,23]. Some mechanisms have been proposed to explain the effects of CAP, namely properties of ultraviolet radiation [24], electric fields that may affect cellular permeabilization by increasing calcium permeability [25], and reactive species that may alter the intracellular redox state, triggering critical cellular responses [20]. Thus, it is crucial to understand the potential of CAP when compared to chemotherapy drugs. Based on the population, intervention, comparison, outcome, and study design (PICOS) criteria, this study aimed to systematically review the literature to determine whether CAP can be as effective as chemotherapy in the treatment of TNBC. Since CAP therapy is not yet a clinically approved treatment, TNBC cell lines and animal models were chosen to compare the cytotoxic effects of CAP with chemotherapeutic agents selected according to ESMO and ASCO guidelines [11,12]. Specifically, doxorubicin, epirubicin, cyclophosphamide, paclitaxel, docetaxel, carboplatin, capecitabine, and olaparib were considered for this study. These chemotherapeutic drugs are the most commonly used in clinical practice. Therefore, the main aim of this systematic review was to answer the PICO question: Is cold atmospheric plasma as effective as chemotherapy in the treatment of triple-negative breast cancer? 

## 2. Methods

This systematic review was developed and reported following the Preferred Reporting Items for Systematic Reviews and Meta-Analyses (PRISMA) guidelines [26], and its protocol was registered in the International Prospective Register of Systematic Reviews—PROSPERO—with the number CRD42023414394. The research question was built according to the PICO methodology, as described in Table 1.

### 2.1. Search Strategy

The literature search was performed in four databases, namely Medline (through PubMed), Web of Science (all databases), Embase, and Cochrane Library. The search formulas used for each database are presented in the Supplemental Materials. No restriction on publication date was applied, and the English, Portuguese, Spanish, and French language filters were used. The search was completed on 13 February 2023. A manual search of the reference lists of relevant studies was performed to find additional potentially relevant studies. The search results were imported to the reference management program Mendeley Reference Manager© v2.80.1 (Mendeley Ltd., London, UK), and duplicate results were removed.

### 2.2. Inclusion and Exclusion Criteria

Two independent reviewers critically assessed the eligibility of studies for inclusion, first by title and abstract and later by evaluating the full text. In case of uncertainty or discrepancies regarding eligibility, a third reviewer was consulted, and a decision was made by consensus. In the eligibility phase, only in vitro and in vivo studies were considered, according to the following inclusion criteria: (1) cell lines and animal models of TNBC; (2) CAP treatment; (3) treatment with chemotherapy drugs selected according to the most recent European and American Society for Medical Oncology Clinical Practice Guidelines (ESMO and ASCO 2021), specifically doxorubicin or epirubicin or cyclophosphamide or paclitaxel or docetaxel or carboplatin or capecitabine or olaparib, and (4) papers whose main goal was to study the cell viability/proliferation of both treatments alone or in combination or in vivo tumor regression, measured as volume or histopathological changes. The exclusion criteria were as follows: (1) other study types, (2) new drugs, (3) resistant cell lines, (4) delivery methods or other formulations, (5) patient samples, (6) non-approved pharmacological combinations, (7) no report of cell viability/proliferation or tumor regression, (8) other experimental models, and (9) other types of studies whose main aim was not to evaluate the efficacy of treatments.

### 2.3. Data Extraction

For studies that met the inclusion criteria, the following information was collected: (1) authors and year of publication; (2) study model (i.e., triple-negative cell line(s) or animal model(s)), (3) chemotherapy treatment (i.e., chemotherapy agent, concentration drug, periodicity of drug administration and/or how it was carried out), (4) CAP treatment (i.e., plasma source, features of machine as voltage, and exposure times), (5) combination treatments with chemotherapy and CAP, (6) methods for cytotoxicity assay and/or histopathological assessment, and (7) cell viability/proliferation and in vivo tumor regression measurements.

### 2.4. Quality Assessment

The risk of bias of the in vitro studies was evaluated with Toxicological Data Reliability Assessment Tool (ToxRTool), which provides guidance on assessing the consistency and quality of toxicologic data [27]. The methodological quality of in vivo studies was checked by assessing the risk of bias with the Systematic Review Centre for Laboratory Animal Experimentation (SYRCLE) risk-of-bias tool [28]. Two independent authors evaluated the quality assessment methodology of eligible studies included in this systematic review.

## 3. Results

### 3.1. Study Selection

A total of 19,364 studies were obtained for analysis, with 4614 articles from PubMed (4507 regarding chemotherapy drugs plus 107 on CAP treatment), 6308 articles from Web of Science (6016 regarding chemotherapy drugs plus 292 on CAP treatment), 7308 articles from Embase (7219 regarding chemotherapy drugs plus 89 on CAP treatment), and 1134 from Cochrane Library (1107 regarding chemotherapy drugs plus 27 on CAP treatment). Before the screening, duplicate articles (8365) were removed using Web Manager (Clarivate™), leading to 10,999 records. Of these, 10,927 were excluded based on title and abstract analysis, resulting in 72 studies for full-text reading. Despite attempts to obtain all full-text records, only 66 were available to assess eligibility. Of these, seven articles were excluded for not meeting the inclusion criteria (two did not examine any drugs included in our review, one performed a mathematical analysis not encompassed in the inclusion criteria, and four did not evaluate cell viability/proliferation as defined in the PICO strategy). Thus, 59 articles were included in this systematic review for full-text reading and analysis, with publication dates from 1986 to 2023.

**Figure 1:**
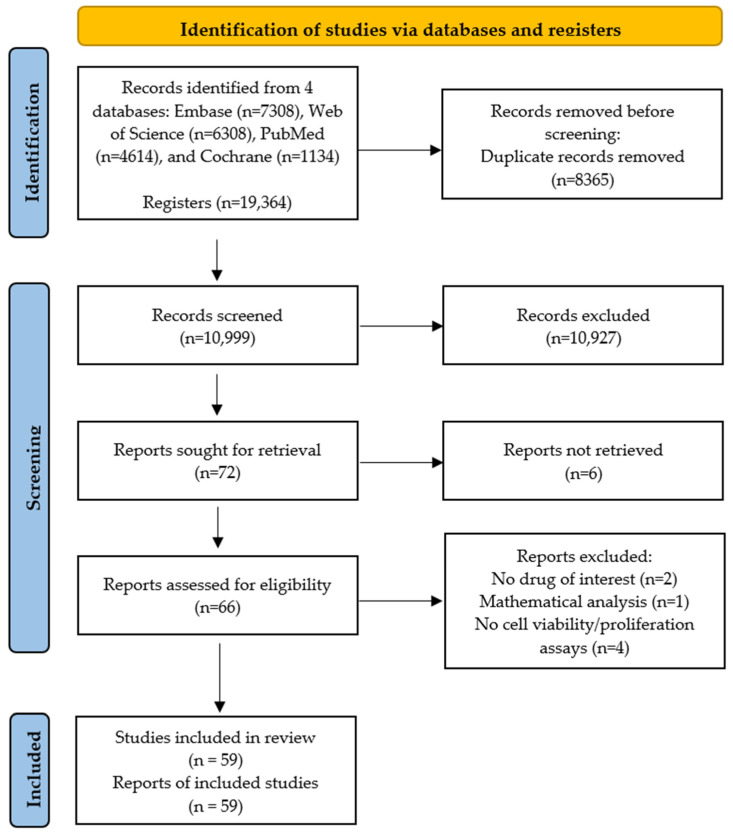
PRISMA flow diagram summarizing study selection in this systematic review.

The PRISMA flow diagram, which summarizes the study selection performed in this systematic review, is shown in Figure 1.

### 3.2. Studies’ Characteristics

Table 2 summarizes the main results observed in the studies included. In this systematic review, 59 studies were considered. Of these, 54 were in vitro studies, 3 were in vivo, and 2 articles included in vitro and in vivo experiments. The outcomes regarding the different types of studies are reported separately. We found articles on all chemotherapy drugs considered, as well as CAP treatment. Concerning CAP therapy, we identified two distinct methods: directly administered CAP and plasma-activated media (PAM), an indirect approach consisting of previously exposed solutions. Moreover, a table quantifying the percentage of inhibition induced by the therapies administered was compiled and included in the Supplemental Materials (Table S1).

#### 3.2.1. In Vitro Studies

The TNBC cell line MDA-MB-231 was employed in most of the research. The BT-20, BT-549, CAL51, CAL148, DU4475, HCC1143, HCC1395, HCC1806, HCC1937, HCC28, HCC38, HCC70, Hs578T, MDA-MB-157, MDA-MB-436, MDA-MB-453, MDA-MB-468, MFM223, MUM51, SUM52, SUM102, SUM149, SUM159, and SUM185 cell lines were also used. MTT [3-(4,5-dimethylthiazol-2-yl)-2,5-diphenyl-2H-tetrazolium bromide], MTS [3-(4,5-dimethylthiazol-2-yl)-5-(3-carboxymethoxyphenyl)-2-(4-sulfophenyl)-2H-tetrazolium], sulforhodamine B (SRB) assay, annexin-V/propidium iodide (PI) for flow cytometry (FC), trypan blue, and clonogenic assay were performed as described in Table 2. Detailed values can be consulted in Table S1.

##### Chemotherapy

Paclitaxel, docetaxel, doxorubicin, olaparib, cyclophosphamide, and carboplatin concentrations ranged from 0.1 nM to 10,000 nM, from 0.1 nM to 500 nM, from 0.1 µM to 100 µM, from 0.001 µM to 250 µM, from 0.01 µM to 1 µM, and from 1 µM to 20 µM, respectively. The concentrations of capecitabine and epirubicin were not reported for in vitro studies [40,66]. Survival, assessed via the formation of colonies after 14 days, was inhibited by paclitaxel concentrations higher than 5 nM [83]. Cell viability was reduced to 80% with the exposure to 6 µM of doxorubicin for 24 hours [30]. The half-maximal inhibitory concentration (IC50) of doxorubicin was 0.3 µM in MDA-MB-231 and BT-20 cells [47,49]; however, other studies reported different values regarding MDA-MB-231 cells (1 µM [67], 6602 nM [64], 888.75 ± 65.26 nM [73], 45–50 µM and 5–10 µM [74]). The concentration of docetaxel that reduced cell proliferation by 75% in MDA-MB-231 cells was 2 nM when a 24-hour incubation was performed [42]. Cyclophosphamide induced a decrease in cell proliferation at 1 μM. Olaparib demonstrated an IC50 > 100 µM and an IC50 = 18 µM in MDA-MB-231 and MDA-MB-468 cells, respectively [37]. However, a different study in MDA-MB-231 and MDA-MB-468 cells reported values of IC50 = 13.5 µM and IC50 = 5.2 µM, respectively [45]. Concentrations from 1 µM to 10 µM (olaparib) were insufficient to induce significant alterations in the cell viability after 72 hours [78]. Carboplatin showed an IC50 = 10 µM after 24 h, and 1, 4, 8, 10, and 20 µM significantly reduced cell proliferation (MTT assay) [60]. The IC50 of capecitabine in MDA-MB-231 cells was 5150 µM and 2790 µM after incubations of 24 and 72 hours [66]. Epirubicin, in combination with other drugs [40,50], namely paclitaxel, demonstrated an antagonistic effect [50]. Doses of carboplatin lower than 10 µM, and paclitaxel showed additive interactions in MDA-MB-231 cells [50]. Despite the vast number of strategies used for chemotherapy studies, doxorubicin, paclitaxel, and docetaxel seemed to be the most promising drugs, exhibiting greater cell viability reduction at lower concentrations. Paclitaxel was capable of inhibiting 50% of cell viability at lower concentrations (0.07 nM–SRB assay) in contrast to olaparib (13.5 µM–MTT assay), with all IC50 values for the MDA-MB-231 cell line being compared.

##### CAP Treatment

This section describes the analysis of 19 studies. Regarding PAM treatment, the solutions used were millipore water, cell culture medium, and Ringer’s solution. A decrease of cell proliferation to 20% (*p* = 0.001) was observed in a volume of 150 µL and 200 µL of PAM (millipore water-based) [72]. The cell viability was reduced to 0.41 and 0.46 in MDA-MB-231 and MDA-MB-468 cells, respectively, compared to control cells after 5 minutes of PAM treatment (medium-based) [79]. The viable cells significantly decreased from 80.50 ± 1.59% to 65.00 ± 3.39% after 120 seconds of CAP treatment in HCC1806 cells [20], while another study demonstrated a reduction of more than 50% in the MDA-MB-231 cell line [52]. Chen et al. reported a reduction of cell viability of approximately 27.4% and 14.7%, respectively, with argon or helium gas flow. CAP decreased cell viability in a dose-dependent manner with statistical significance, as described in Ma et al. [55]. The viability of the MDA-MB-231 cell line decreased to under 40% or 50% after 5 minutes of CAP or PAM exposure, respectively [44]. Furthermore, apoptosis seemed to be the most prevalent type of cell death [20,55,65]. In general, CAP and PAM demonstrated an effective anti-tumoral effect in short exposure times, such as 60 and 120 seconds; however, some authors tested longer exposure times, mainly in PAM treatment.

##### CAP Treatment and Chemotherapy

Interestingly, CAP treatment and chemotherapy drugs, specifically paclitaxel and olaparib, were combined in two studies [19,51]. Olaparib showed a tendency to improve the efficacy of CAP in all cell lines [51]. Moreover, the chemosensitivity to paclitaxel (0.01 µM) was improved after 15 seconds of CAP exposure. According to clonogenic assay, the combined treatment decreased the number of colonies to a number similar to or even smaller than that specific to PAM or paclitaxel [19].

#### 3.2.2. In Vivo Studies

To obtain animal models, the strains CB-17 of severe combined immunodeficiency mice [56,61,70] and Balb/c were used [79,84] and were inoculated with the TNBC cell line MDA-MB-231.

##### Chemotherapy

As described by Man et al., cyclophosphamide was administered following two regimens. The group continuously administered 25 mg/kg of cyclophosphamide (low concentration) via drinking water showed an initial reduction of tumor size with no weight loss for 50 days. However, the group of mice treated in cycles of 6 days with 450 mg/kg/cycle (150 mg/kg/injection every other day) demonstrated severe weight loss and death one week after starting the therapy [56]. Munõz et al., demonstrated that 20 mg/kg/day via drinking water starting on the 14th day reduced the volume of tumor compared to the control group. The percentage of necrosis increased from the control group (78%) to the treated group (85%), and no weight loss or other signs of toxicity were observed [61]. Another study demonstrated that cyclophosphamide (20 mg/kg/day) added to capecitabine (100 mg/kg) significantly increased survival compared to the control [70].

##### CAP Treatment

CAP treatment was administered directly into animal tumors, and PAM injection of PBS showed significant inhibition of tumor growth (*p* = 0.044 and *p* = 0.017, respectively). In the comparison of both approaches, the survival of mice in the CAP treatment group was significantly higher than that in the PAM group (*p* = 4.9 × 10^−4^). In addition, all control mice died within 27 days, while all mice from the CAP group survived until the end of the experiment (30 days) [84]. Another study showed that the tumor volume was inhibited by PAM injection, and tumor weight dropped considerably after treatment (from 4.053 g to 0.787 g, *p* = 4.69 × 10^−4^). No visible adverse effects were observed [79].

### 3.3. Quality Assessment

**Figure 2:**
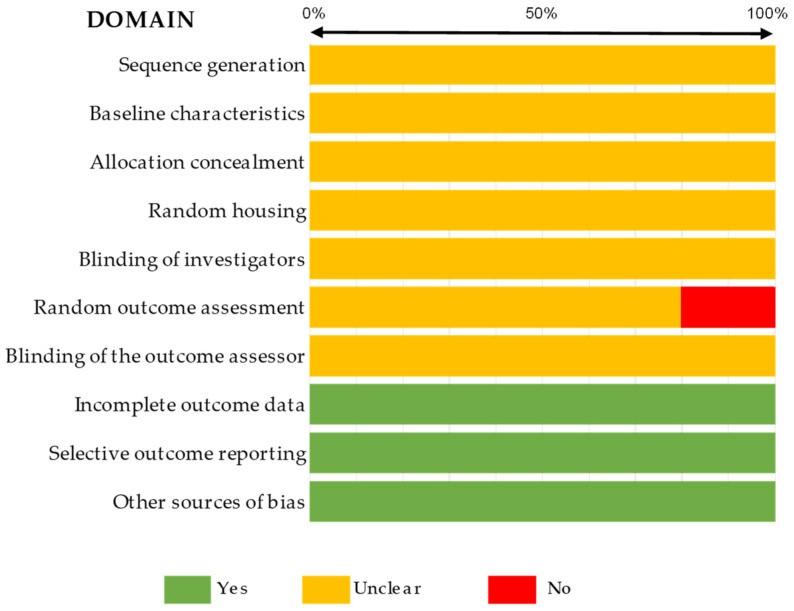
Summary of the quality assessment of the in vivo studies included in the systematic review completed with the SYRCLE tool.

**Figure 3:**
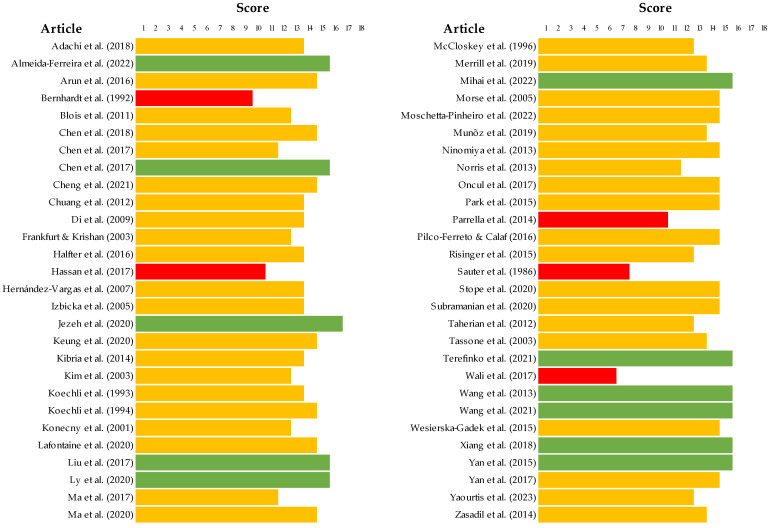
Score of the quality assessment of the in vitro studies included in the systematic review completed with the ToxRTool [19,20,21,29,30,31,32,33,34,35,36,37,38,39,40,41,42,43,44,45,46,47,48,49,50,51,52,53,54,55,57,58,59,60,61,62,63,64,65,66,67,68,69,71,72,73,74,75,76,77,78,79,80,81,82,83]. Articles with scores <11 were represented in red, scores between 11–14 were represented in yellow, and scores between 15–18 were represented in green.

In terms of quality assessment, most studies presented an unclear bias, determined by the SYRCLE tool regarding in vivo studies, as described in Figure 2 and detailed in Supplemental Table S2. According to these bias tool guidelines, only three parameters were correctly reported for all studies, specifically, incomplete outcome data, selective outcome reporting, and other sources of bias. Moreover, 20% of articles presented no data on random outcome assessment, as shown in Figure 2. For other studies, the ToxRTool protocol was used, and articles were categorized based on this score. We observed scores between 11 and 14 as the most prevalent, where the studies were considered reliable with restrictions. Although five articles were classified as not reliable, eleven studies were classified under the criterion of reliability without restrictions. The risk assessment for individual study bias is represented in Figure 3 and detailed in Supplemental Table S3.

## 4. Discussion

The clinical complexity of treating TNBC often requires a tailored approach due to its aggressive nature and poor prognosis compared to other molecular subtypes [85,86,87]. Chemotherapy is a well-established treatment indicated in clinical practice regarding TNBC [10,11]; however, it has associated adverse effects. CAP has been investigated across a vast array of medical fields, specifically in dental medicine, regeneration of tissues, and tumor therapy, without causing significant harm to healthy cells, as well as having antimicrobial effects and an impact on stem cells and nitric oxide levels [88]. It has been explored as a new emerging medical approach for several types of cancer, including TNBC, with promising results in terms of cell death [20,44,75,77]. As CAP is not a clinically approved treatment, being under preclinical research, a comparison was conducted to determine the efficacy of both treatments without associated interventions. 

The chemotherapeutic drugs explored—paclitaxel, docetaxel, cyclophosphamide, doxorubicin, olaparib, carboplatin, and capecitabine—were selected based on ESMO and ASCO guidelines [11,12], ensuring a broad and complete assessment of cellular effects [52,79]. Currently, chemotherapy drugs are used in clinical practice and are heavily used as a positive control in most experiments [89,90]. For all treatments, the outcomes depended on the protocol definitions, including the dose of chemotherapy drugs administered and the time of exposure to CAP. Several assays, such as MTT, MTS, SRB, annexin-V/propidium iodide, trypan blue, and clonogenic assay, were performed to prove the in vitro efficacy of CAP and chemotherapy drugs.

We found a wide range of chemotherapy concentrations tested in in vitro assays. The papers showed a reduction in cell proliferation, which is supported by the mechanisms of action of the drugs on tumor cells [91,92,93,94,95]. There were additive interactions between paclitaxel and carboplatin in vitro [50]. In fact, in patients whose TNBC disease progressed after taxane administration, carboplatin was one of the recommended chemotherapy agents [11,12]. According to the guidelines, taxane-anthracycline-based combinations are options for treatment [11,12]. The results obtained corroborated our expectations about the efficacy of chemotherapy in TNBC cell lines. However, the evidence available was often insufficient to statistically compare several studies since the data were not described quantitatively, and the various strategies do not allow for a comparative evaluation. 

CAP therapy is an emerging therapeutic approach in cancer although the mechanism of action remains unclear. Recent studies have shown its effects on cell proliferation in several types of cancer, leading to cell death [35,44,96,97,98]. Here, we examine two different strategies concerning the application of CAP in TNBC cell lines, namely CAP and PAM, which used different exposure times and solutions. Both strategies are also reported in preclinical studies regarding other types of cancer [17,18,19,44,99,100,101]. Different types of equipment that are able to generate CAP are described. Some authors used a flow of gases such as argon or helium [34,44,53,62,77], and the frequency and high voltage were also dependent on the equipment of each research group. Generally, the exposure times were 60 seconds or 120 seconds [19,20,21,33,35,44,51,52,53,55,71,80,81]. The reduction of cell viability in TNBC cells was time dependent in all studies. Apoptosis seemed to be the predominant type of cell death [19,20]. Studies on different types of cancer demonstrated a close correlation with the abovementioned, supporting the anti-tumoral potential of CAP as a promising strategy. A vast number of cell lines representing melanoma, brain tumor and leukemia, cervical, breast, colorectal, gastric, lung, ovarian, head and neck, and pancreatic cancers have already been used for CAP studies, demonstrating its anti-proliferative effect [88]. Moreover, some authors reported the selectivity of CAP to tumor cell lines [22,102,103]. The efficacy of olaparib or paclitaxel combined with CAP tended to improve the cytotoxicity of treatment in five distinct TNBC cell lines [51]. PAM improved the chemosensitivity to the lowest concentration of paclitaxel and reduced the metabolic activity compared to isolated therapies [19]. 

Translating cell line models into animal models is a crucial stage in assessing the efficacy of therapy in a biologically complex organism. In the studies, the methods used were tumor growth or volume, animal weight, or survival monitoring. Cyclophosphamide was able to reduce the volume of tumor with no weight loss and no signs of toxicity [61]. The combination of cyclophosphamide and capecitabine increased survival [70]. Similarly, CAP and PAM inhibited tumor growth significantly, and no noticeable adverse effects were observed [52,84]. CAP was applied locally to the tumor in both plasma approaches. Although we only analyzed two papers with CAP treatment in vivo, the results proved the efficacy in TNBC. In vivo studies with different types of cancer have been performed in recent years [104,105]. Nevertheless, further studies should include cell lines representative of other TNBC molecular subtypes and consider other animal models to encompass as many of the characteristics observed in clinical practice as possible. Unfortunately, in vivo studies regarding CAP and chemotherapy combination are unavailable. In the future, to maximize the anti-tumor potential of CAP, combining it with chemotherapy drugs should also be studied in vivo, promoting a potential coordinated translation to the clinical setting. Furthermore, patient-derived xenograft models could be an enriched research option for evaluating CAP efficacy in human BC tissue samples.

As regards CAP, it could be a promising option for clinical practice. From the results obtained, we hypothesize a reduction of the side effects associated with chemotherapy with a lowering of the concentrations of the drugs administered.

One of the limitations observed was the heterogeneity between the studies due to methodological approaches, namely exposure, intervals and doses of drugs, voltages of equipment, times of exposure, and evaluation times. Moreover, the lack of important information, such as concentrations and detailed and quantitative results, impeded meta-analysis studies. In addition, we observed differences between the number of studies for each drug, which means that the sample included in some groups was reduced, limiting the conclusions. The bias assessment, based on a set of well-defined guidelines, proved the difficulty in obtaining all the information necessary to proceed to the planned statistical study, as illustrated in Figure 2 and Figure 3. CAP studies provided a more detailed description of the conditions of treatment used. Table 3 summarizes key aspects of this systematic review, including the main considerations of the study and the next steps. In the future, based on our results, we suggest creating a list of standard methodologies to address the limitations found. Moreover, correlating studies with human tissue samples of TNBC after CAP treatment with clinical reports of patients will enhance the potential translational value of therapy. Nevertheless, combining chemotherapy and CAP or PAM in vivo appears to be a strategic option.

## 5. Conclusions

Chemotherapy agents effectively reduced cell proliferation in most TNBC cell lines, depending on a wide range of concentrations and experimenting conditions. CAP treatment successfully treated TNBC cell lines, with apoptosis being the most prevalent type of cell death. Paclitaxel or olaparib combined with CAP in vitro should be further investigated. Our results suggest that other combinations should be considered and evaluated. In the in vivo studies, the selection of different chemotherapeutic regimens influenced the obtained results, with both CAP and PAM effectively reducing tumor volume without visible side effects. However, the lack of information on both treatments and the diversity of experimental conditions underscore the need for more research. Despite the weaknesses observed, our results indicate that CAP is as effective as chemotherapy in TNBC. Innovative studies should be performed to increase the knowledge of CAP treatment and enhance future medical options on TNBC.

## Figures and Tables

**Table 1:** Population, intervention, comparison, and outcome (PICO) research strategy used in this systematic review.

Parameter	Description
Population (P)	Triple-negative breast cancer cell lines and animal models
Intervention (I)	Cold atmospheric plasma
Comparison (C)	Doxorubicin or epirubicin or cyclophosphamide or paclitaxel or docetaxel or carboplatin or capecitabine or olaparib
Outcome (O)	In vitro studies: cell viability/proliferation In vivo studies: tumor volume and histopathological changes

**Table 2:** Studies’ details and outcomes.

Author (Year)	Type of Study	Experimental Model	Treatment Characteristics	Methods	Main Results
Adachi et al. (2018) [29]	In vitro	MDA-MB-231	PTX (1, 10, 100 nM, and 1 µM) Incubation: 48 h PTX (100 nM) or DOX (10 µg/mL) for FC Incubation: 24 h	Viable cells counted with a hemocytometer; Annexin V/PI (FC)	The growth of cells in vitro was significantly inhibited by increasing doses of PTX (*p* < 0.01). Cells treated with PTX (100 nM) for 24 h showed a slight increase in early apoptotic cells. DOX markedly inhibited cells in a dose-dependent manner (*p* < 0.01). Apoptosis analysis confirmed an apparent increase in early plus late apoptotic cells 24 h after DOX^3^ treatment.
Almeida-Ferreira et al. (2022) [20]	In vitro	HCC1806	CAP. Plasma device: 4 kV, pulses of 1 kHz through a sterilized needle 0.9 mm in radius and 40 mm in length Exposure time: 60 and 120 s Incubation: 24 h	Annexin V/PI (FC)	The proportion of viable cells significantly decreased from 80.50 ± 1.59% to 64.67 ± 2.16% (*p* = 0.0008) after 60 s of exposure and to 65.00 ± 3.39% (*p* = 0.01) after 120 s.
Arun et al. (2016) [30]	In vitro	MDA-MB-231	DOX (1, 2, 3, 4, 5, and 6 µM) Incubation: 12 and 24 h	MTT assay	DOX (1 μM) resulted in 90% of cell viability, and DOX (6 μM) resulted in 80% at 12 h. Cell viability was 65% after 24 h when low doses were used.
Bernhardt et al. (1992) [31]	In vitro	MDA-MB-231	DOX (1 µM) Incubation: 0–333 h	Crystal violet assay	The drug was cytotoxic (data were not shown).
Blois et al. (2011) [32]	In vitro	MDA-MB-231	PTX (100 mM) Incubation: 48 h	SRB assay	IC50 (nM): 0.07
Chen et al. (2018) [33]	In vitro	MDA-MB-231	CAP. Plasma device: 8 kV, 16 kHz. He gas was injected into the quartz tube with a 0.2 L/min flow rate. Micro-sized CAP with stainless-steel tubes 20 mm and 60 mm in length. Exposure time: 5, 10, 30, 60, and 120 s Incubation: 24 and 48 h	MTT assay	The cell viability dropped with increasing treatment time for both 20 mm and 60 mm µCAP treatment. For each exposure time, the cell viability of breast cancer cells was lower for the 20 mm CAP length than for the 60 mm CAP length.
Chen et al. (2017) [34]	In vitro	MDA-MB-231	PAM. Plasma device: immersed in DI water. The electrodes were connected to a secondary high-voltage transformer (2–5 kV, 30 kHz). Ar, He, and N_2_ were used, and the flow rate was maintained at about 0.3 min^−1^. Exposure gas: Ar, He, and N_2_ Solutions: DI water and DMEM Incubation: 24 and 48 h	MTT assay	After 24 h, cell viability decreased by approximately 27.4% and 14.7% when treated with Ar and He plasma, respectively. Only a slight decrease in cell viability was observed in the case of DI water and N_2_ plasma solution. After 48 h, viability decreased approximately 73.1%, 22.8%, 14.1%, and 13.5% when cells were treated with Ar plasma solution, He plasma solution, N_2_ plasma solution, and DI water, respectively. Thus, the most potent effect was observed in the case of Ar plasma, while the smallest was in N_2_ plasma.
Chen et al. (2017) [35]	In vitro	MDA-MB-231	PAM. Plasma device: 1–3012 V, 40 mA activated with low or high current Solutions: DI water and DMEM Exposure time: 12, 24, 36, 48, and 60 s Incubation: 24 and 48 h	MTT assay	The viability of cells incubated in low-current PAM was generally lower than that of cells incubated in high = current PAM. This was observed both after 24 h (36 s, *p* > 0.05; 48 s, *p* < 0.05; 60 s, *p* < 0.001) and 48 h (36 s, *p* < 0.01; 48 s, *p* > 0.05; 60 s, *p* < 0.05). The cell viability of cells incubated for 48 h at low current steadily decreased with treatment duration, while the cell viability at high current initially decreased and then increased slightly.
Cheng et al. (2021) [36]	In vitro	MDA-MB-231	CAP. Plasma device: Canady Helios Cold Plasma™. He flow rate at 3 L/min; 4 kV, 300 kHz, and 40 W Power settings: 80P (15.7 W), 100P (22.3 W) and 120P (28.7 W) Exposure time: 3, 5, and 6 min Incubation: 6, 24, and 48 h	Annexin V/PI (FC)	Exposure for 3, 5, or 6 min at 80P or 120P reduced the live cells after 24 and 48 h of incubation.
Chuang et al. (2012) [37]	In vitro	MDA-MB-231, MDA-MB-468, CAL51	Olaparib (20, 40, 60, 80, or 100 µM) Incubation: 72 h	MTT assay; Clonogenic assay (14–21 days)	IC50 (µM) by MTT MDA-MB-231: >100; MDA-MB-468: 18; CAL51: 9.5 IC50 (µM) by clonogenic assay MDA-MB-231: 4.5; MDA-MB-468: 0.2; CAL51: 0.4
Di et al. (2009) [38]	In vitro	MDA-MB-231	DOX	Clonogenic assay (14 days)	The mean number of colonies was 2.7 ± 0.9% compared to the control.
Frankfurt & Krishan (2003) [39]	In vitro	MDA-MB-468	DOX or PTX Incubation: 48 h	MTT assay SRB assay	IC50 (µM) by MTT and SRB assays, respectively. DOX: 0.05; 0.1 PTX: 0.01; 0.01
Halfter et al. (2016) [40]	In vitro	HCC1143 and HCC1937 spheroids	Single or combined regimens of CAR, CCP, DOC, EPI, and PTX Incubation: 96 h	ATP assay	Metabolic activity (HCC1143 and HCC1937, respectively) CAR: 104.71 ± 26.68; 80.95 ± 0.67 DOC: 121.36 ± 45.60; 101.38 ± 2.69 PTX: 81.97 ± 42.22; 93.37 ± 1.71 EPI and CCP: 86.07 ± 24.99; 85.12 ± 5.26 EPI and CCP and DOC: 117.00 ± 23.75; 87.57 ± 5.03 EPI and CCP and PTX: 91.59 ± 33.35; 83.74 ± 10.44 DOC and DOX and CCP: 97.56 ± 6.57; 75.06 ± 2.80
Hassan et al. (2017) [41]	In vitro	MDA-MB-436, MDA-MB-231, MDA-MB-453, MDA-MB-468, HCC1143, HCC1937, HCC1806, HCC1395	Olaparib (0.25 nmol/L to 100 mmol/L) Incubation: 9 days, drugs plus media changed after 4–5 days	Chemosensitivity assay	IC50 differed in all cell lines, ranging from 0.003 to 3.8 mmol/L.
Hernández-Vargas et al. (2007) [42]	In vitro	MDA-MB-231	DOC (from 0 to 500 nM) Incubation: 24–96 h	Crystal violet assay; Annexin-V-FITC Apoptosis detection kit (FC)	Cells were sensitive to nM concentrations of DOC. There was a growth inhibition at concentrations lower than 10 nM. IC75 (nM): 2 nM
Izbicka et al. (2005) [43]	In vitro	MDA-MB-231	DOC (0.1, 0.5, or 5 nM) or PTX (0.1, 1, or 5 nM) Incubation: 72 and 120 h	MTS assay	IC50 (pM) DOC: 499 (72 h); 35 (120 h) PTX: 933 (72 h) PTX toxicity at day five increased about twofold in comparison with day three.
Jezeh et al. (2020) [44]	In vitro	MDA-MB-231	CAP or PAM. Plasma device: 20–70 kHz and 5 kV. Gas flow: He or He + 0.5% O_2_ Exposure time: 1, 2, 3, 4, and 5 min Incubation: 48 h PAM: 200 µL of medium	MTT assay	CAP: Cell viability was reduced in almost all CAP exposure. Generally, better results were obtained using He + 0.5% O_2_ than pure gas. The viability of MDA-MB-231 cells decreased by more than 60% after 5 min of treatment. PAM: Similar results were observed despite no significant differences between He + 0.5% O_2_ and He. Cell viability decreased to about 50% after 5 min.
Keung et al. (2020) [45]	In vitro	MDA-MB-231, MDA-MB-436, MDA-MB-468, HCC1143, HCC1937, BT-549, HCC70, HCC1806	Olaparib (from 0.001 to 200 μM) Incubation: 7 days	MTT assay	IC50 (µM) MDA-MB-231: 13.5; MDA-MB-436: 4.7; MDA-MB-468: 5.2; HCC1143: 14; HCC1937: 96; BT-549: 81; HCC70: 11; HCC1806: 1.2
Kibria et al. (2014) [46]	In vitro	MDA-MB-231	DOX (several concentrations) Incubation: 24 h with medium changed after 8 h	WST-8 assay	EC50: 25.72 ± 20.27 μg/mL
Kim et al. (2003) [47]	In vitro	MDA-MB-231	DOX or PTX Incubation: 48 h	MTT assay	IC50 (µM) DOX: 0.3 PTXl: 0.03
Koechli et al. (1993) [48]	In vitro	BT-20	PTX (0.001; 0.002; 0.005; 0.01 PPCs) or DOX (0.1; 0.2; 0.5; 1.0 PPCs) or PTX and DOX (1:1000)	ATP cell viability assay	IC50 (PPCs): PTX: 0.00163; DOX: 0.319; PTX and DOX: 0.2277. The CI values ranged from 5.4 to 0.9. At a ratio of 1:10 (PPC), the CI values ranged from 0.4 to 0.5, indicating synergism over the whole range.
Koechli et al. (1994) [49]	In vitro	BT-20	DOX or PTX or CCP (0.01, 0.02, 0.05, 0.1, and 0.5 PPC	ATP cell viability assay	IC50 (µM) DOX: 0.32; PTX: 0.007; CCP: 5.53
Konecny et al. (2001) [50]	In vitro	MDA-MB-231	PTX (0.9, 1.8, 3.6, 7.2, 14.5, 29, 58, 116 nM) and CAR (3.1, 6.2, 12.5, 25, 50, 100, 200, 400 µM) or PTX (0.4, 0.9, 1.8, 3.6, 7.2, 14.5, 29, 58, 116 nM and EPI (1.7, 3.3, 6.7, 13.4, 26.8, 53.7, 107, 215, 430 nM)	Crystal violet assay	CAR doses (<10 μM) showed additive interactions in combination with PTX. However, EPI and PTX demonstrated an antagonistic effect.
Lafontaine et al. (2020) [51]	In vitro	BT549, Hs578T, MDA-MB-157, MDA-MB-231, MDA-MB-468	CAP. Plasma device: 10 or 35 W, He and O_2_ gas flow Exposure time: 10 to 120 s Olaparib Concentration: 2 µM Incubation: 2 h before CAP	Crystal violet assay	CAP: Only 30 s of CAP treatment reached a more intense effect than did other application modes. The efficacy increased with treatment time. Olaparib: Affected cell growth, especially for MDA-MB-468 (more than 60% of inhibition, *p* < 0.001). CAP + olaparib: The combination improved the cytotoxic effect of CAP in all cell lines.
Liu et al. (2017) [52]	In vitro	MDA-MB-231, MDA-MB-453	CAP. Plasma device: 10 kV and 5 mA Exposure time: 60, 90, and 120 s Incubation: 48 h	Trypan blue	There was significant reduction of cell viability after 60 s in MDA-MB-231, while MDA-MB-453 did not show significant reduction. After 120 s, CAP treatment decreased the viability to <80% MDA-MB-453 and <50% MDA-MB-231.
Ly et al. (2020) [53]	In vitro	MDA-MB-231, Hs578T, HCC1806	CAP. Plasma device: Canady Helios Cold Plasma™ Scalpel, 4 kV, He flow rate at 3 L/min and power set to 80, 100, and 120 P. Exposure time: 1, 2, 3, 4, 5, and 6 min Incubation: 48 h	MTT assay	Increasing power and treatment duration from 80 to 120 P for 1–6 min yielded a greater viability reduction in MDA-MB-231. A 92–99% decrease in cell viability was achievable after 120 P at 5 or 6 min (*p* ≤ 0.05). HCC1806 showed the greatest overall CAP resistance.
Ma et al. (2017) [54]	In vitro	HCC1937, BT-549, HCC38	PTX	Annexin V/PI (FC) and 7AAD kit (BD559763)	PTX increased the number of apoptotic cells.
Ma et al. (2020) [55]	In vitro	MDA-MB-231	CAP. Plasma device: 12 kV, 24 kHz. Power density: 0.9 W/cm^2^. Gas flow: He at 120 L/h Exposure time: 30, 60, 90, and 120 s Incubation: 24 h	Cell counting kit-8 kit and Annexin V-FITC/PI (FC)	CAP significantly decreased the cell viability in a dose-dependent manner and induced apoptotic cell death.
Man et al. (2002) [56]	In vivo	MDA-MB-231; Female CB-17 SCID mice; Orthotopically implanted into the mammary fat pad	CCP (25 mg/kg of continuous low doses via drinking water; 450 mg/kg/cycle: 150 mg/kg/injection every other day over six days)	Tumor and weight monitoring	Six-day therapy cycles were similar to low-dose administration for tumor size reduction. However, the former was extremely toxic to SCID mice, resulting in severe weight loss and death of mice after the first week. No weight loss or other signs of toxicity were observed in the group of SCID mice treated via drinking water.
McCloskey et al. (1996) [57]	In vitro	MDA-MB-468	PTX Incubation: 3, 24, and 120 h	Trypan blue	Cells exposed for 3 h demonstrated concentration-dependent growth inhibition at ≥10 nM PTX (IC50 = 17 nM). At 24 h, growth inhibition was at 1 nM (IC50 = 2.6 nM). At 120 h, the IC50 was 1.8 nM.
Merrill et al. (2019) [58]	In vitro	MUM51, BT-20, BT-549, CAL148, CAL51, DU4475, HCC1806, HCC1937, HCC38, HCC70, Hs578T, MDA-MB-157, MDA-MB-231, MDA-MB-436, MDA-MB-453, MDA-MB-468, MFM223, SUM52, SUM102, SUM149, SUM159, SUM185	PTX (from 0.6 to 10,000 nM) or DOC (from 0.1 to 1000 nM) Incubation: 72 h	WTS-1 followed by CellTiter-Glo^®^ assay	IC50 (nM) PTX: 110 (MUM51); 159 (BT-20); 110 (BT-549); 4 (CAL148); 310 (CAL51); 19 (DU4475); 77 (HCC1806); 130 (HCC1937); 1700 (HCC38); 3 (HCC70); 150 (Hs578T); 190 (MDA-MB-157); 200 (MDA-MB-231); 110 (MDA-MB-436); 2 (MDA-MB-453); 89 (MDA-MB-468); 4 (MFM223); 9 (SUM102); 13 (SUM149); 2 (SUM159); 10 (SUM185); 3 (SUM52). DOC: 2 (MUM51); 2 (BT20); 1 (BT549); 2 (CAL148); 4 (CAL51); 5 (DU4475); 4 (HCC1806); 1 (HCC1937); 1 (HCC38); 1 (HCC70); 1 (Hs578T); 1 (MDA-MB-157); 2 (MDA-MB-231); 1 (MDA-MB-436); 1 (MDA-MB-453); 1 (MDA-MB-468); 740 (MFM223); 1 (SUM102); 5 (SUM149); 140 (SUM159); 2 (SUM185); 2 (SUM52).
Mihai et al. (2022) [19]	In vitro	MDA-MB-231 and MDA-MB-231 spheroids	PAM. Plasma device: 10 kV and 28 kHz Solution: DMEM without FBS (160 µL) Exposure time: 30 and 60 s Incubation: 20 min and medium changed to 10% FBS DMEM PTX (0.1 μM, 0.01 μM, and 0.001 μM) after PAM Incubation: 24 and 48 h	MTT assay; Clonogenic assay; Spheroid area	PTX: Cell viability was reduced to 63.05% and 28.31% (0.1 μM PTX) after 24 and 48 h, respectively. PAM: At 48 h, cell viability reduced to approximately 25%. PTX + PAM: Cell line showed values between 33.77% and 36.28% at 24 h and 18.80% and 19.95% at 48 h. After 15 s of PAM, the total area of spheroids significantly decreased to 23.81% compared to control (*p* < 0.05) and 20.95% compared to PTX treatment. Cells were susceptible to PAM and combined treatment. PAM could induce a stable cytotoxic effect and improve PTX chemosensitivity.
Morse et al. (2005) [59]	In vitro	MDA-MB-231	DOC (10 nmol/L) (1) Incubation: 48 h (2) Incubation: 24 or 48 h or 48 h followed by 24 h in drug-free medium (3) Incubation: 0, 2, 4, 8, 16, 24, and 48 h	(1) Trypan blue (2) Crystal violet assay (3) Annexin V/PI (FC)	(1) Cells had 10% lower viability. (2) IC50 (24 h): 9.28 × 10^−8^ (1.63 × 10^−9^ to 5.28 × 10^−6^); IC50 (48 h): 5.12 × 10^−8^ (3.25 × 10^−8^ to 8.07 × 10^−8^); IC50 (48 + 24 h): 5.00 × 10^−8^ (3.43 × 10^−8^ to 7.29 × 10^−8^). (3) The maximal increase of apoptosis was 0.97% (*p* = 0.39) at 8 h.
Moschetta-Pinheiro et al. (2022) [60]	In vitro	MDA-MB-468	CAR (1, 2, 4, 8, 10, and 20 µM) Incubation: 24 h	MTT assay	IC50 (µM): 10 Results showed that within 24 h, all CAR concentrations, except for 2 μM, were able to significantly reduce cell viability when compared to control (*p* < 0.05).
Munõz et al. (2019) [61]	In vitro and in vivo	(1) MDA-MB-231/LM2-4 (metastatic variant) (2) Female CB-17 SCID mice; 2 × 10^6^ cells orthotopically implanted into the right inguinal mammary fat pad	CCP (1) 0.01, 0.05, 0.1, and 1 μM Incubation: 6 days (2) 20 mg/Kg/day through the drinking water, initiated on day 14	(1) MTS assay (2) Tumor growth and volume (mm^3^) and H&E	(1) 1 μM showed a marked decrease in cell proliferation. (2) Tumor volume was reduced. The median of necrosis was 78% (70–80% range) for the control group and 85% (80–90% range) for the treated group. In the invasive tumor border, the percentage of necrosis was 16% (0–40% range) for untreated tumors and 40% (20–80% range) for the CCP group. No weight loss or other signs of toxicity were observed.
Ninomiya et al. (2013) [62]	In vitro	MDA-MB-231	CAP. Plasma device: 9 kHz, He gas flow Exposure conditions: 4, 8, 12, 16, or 18 kV for 600 s Incubation: 24 h	Trypan blue	The half-maximal effective peak-to-peak voltage was 16.7 ± 0.3 kV. Cell viability reduced with the increase in voltage.
Norris et al. (2013) [63]	In vitro	HCC1937	Olaparib (0.02–100 μM) Incubation: 120 h	SRB assay	IC50 (μM): ≈100
Oncul et al. (2017) [64]	In vitro	MDA-MB-231	DOX (50, 100, 200, 400, 800, 1000, 1500, 2000, 3000, 4000, 8000 nM) Incubation: 48 h	SRB assay; Annexin V/PI (FC)	IC50 (nM): 6602 Cells underwent apoptosis in proportions of 6.75, 15, and 8.25% when treated with 50, 200, and 800 nM of the drug, respectively. Necrotic cells increased by 29% as a response to treatment of 800 nM.
Park et al. (2015) [65]	In vitro	MDA-MB-231	CAP Exposure time: 30 s, 10 times	Cell counting kit-8 and clonogenic assay; Annexin V/PI (FC)	Six days after the treatment, CAP reduced the growth rate compared to control. Apoptosis increased from 7.67 to 13.8%.
Parrella et al. (2014) [66]	In vitro	MDA-MB-231	CAB or DOX Incubation: 48 and 72 h	MTT assay	IC50 (µM) CAB: 5150 (24 h) and 2790 (72 h) DOX: 19 (24 h) and 4 (72 h)
Pilco-Ferreto & Calaf (2016) [67]	In vitro	MDA-MB-231	DOX (1, 2, 4, and 8 μM) Incubation: 24 and 48 h	MTT assay	IC50 (μM): 1 The increase in DOX concentration decreased the viability in a time- and dose-dependent manner.
Risinger et al. (2015) [68]	In vitro	MDA-MB-231, MDA-MB-468, BT-549, Hs578T, HCC1937	PTX or DOC	SRB assay	MDA-MB-468 was the most sensitive, and BT-549 and HCC1937 were the most resistant.
Sauter et al. (1986) [69]	In vitro	BT-20	CCP or DOX	Phase-contrast microscopy	Cytotoxicity effect was measured by cytopathogenic effect, with no results from CCP. It was 1.7 μM regarding DOX.
Shaked et al. (2016) [70]	In vivo	MDA-MB-231/LM2–4 (metastatic variant); Female CB-17 SCID mice; 2 × 10^6^ cells were orthotopically implanted in the mammary fat pad of 6-week-old females	CAB (LDM: 100 mg/kg; MTD: 400 mg/kg/day for 4 days followed by a 17-day drug-free break period) or CCP (20 mg/kg/day through the drinking water)	Survival	There were no significant differences in the mortality between CAB LDM, CAB MTD, and control. Adding CCP to the LDM of CAB significantly increased the survival percentage compared to the control (*p* = 0.006).
Stope et al. (2020) [71]	In vitro	MDA-MB-231	CAP Exposure time: 5, 20, and 60 s PAM Exposure time: 20 and 60 s Incubation: 4, 24, 48, 72, 96, and 120 h	Cell counting	CAP: Effects were seen after 20 s of treatment and 72 h of incubation. More pronounced effects were seen after 60 s. After 60 s, a 4.5-fold growth reduction occurred from 4 to 120 h of incubation. PAM: The results showed a slightly lower anti-proliferative potential for PAM than for CAP. From 4 to 120 h, cell growth was reduced threefold. At 48 h of incubation, the cell growth reduced significantly from the control at two conditions.
Subramanian et al. (2020) [72]	In vitro	MDA-MB-231	PAM. Plasma device: 5 kV, 15 kHz, 6.8 ± 0.6 W Solution: UP water Exposure time: 6, 12, and 18 min Volume: 60, 80, 100, 150, and 200 µL for 6 min	MTT assay	Cell viability was 81% (*p* < 0.001), 55% (*p* < 0.001), and 24% (*p* < 0.001) after 6, 12, and 18 min, respectively, under a volume of 200 μL. A significant reduction of cell viability was observed only at higher volumes (>100 μL), with 66% (*p* < 0.01) and 20% (*p* < 0.001) at 150 and 200 μL, respectively.
Taherian et al. (2012) [73]	In vitro	MDA-MB-231	DOX or DOC; Incubation: 48 h	MTT assay	IC50 (nM) DOX: 887.75 ± 65.26; DOC: 634.58 ± 92.4
Tassone et al. (2003) [74]	In vitro	MDA-MB-231, HCC1937	DOX (from 0.1 to 100 µM) or PTX (from 0.01 to 2 µM); Incubation: 48 h	MTT assay; Annexin V/PI (FC)	IC50 (µM) DOX: 45–50 (HCC1937); 5–10 (MDA-MB-231) PTX: 2 (HCC1937); 0.01–0.02 (MDA-MB-231) An apoptotic effect was seen in HCC1937 cells exposed to PTX IC50.
Terefinko et al. (2021) [75]	In vitro	MDA-MB-231	PAM. Plasma device: 6 kV, 66.45 kHz, He gas flow rate at 10.6 L/min Solution: DMEM or Opti-MEM media with or without 3% FBS; Incubation: 24 and 48 h Exposure time: 150, 180, 210, and 240 s Volume: 1.5 or 3 mL	MTT assay; Annexin V/PI (FC)	Without FBS: No significant results in cells incubated with DMEM-activated media. However, after 48 h, Opti-MEM-activated media exhibited a great impact on the decrease in cell viability, especially in the treatment times of 180 and 240 s (** *p* < 0.001, *** *p* < 0.0004, respectively). With FBS: DMEM-activated media did not affect cell viability. On the other hand, Opti-MEM-activated media affected cell viability after 180 and 240 s (** *p* < 0.0014; *** *p* < 0.0002, respectively) at 24 h. At 48 h, cell viability was reduced in all exposure times (* *p* < 0.013; ** *p* < 0.0014; *** *p* < 0.0002). Opti-MEM-activated media exhibited the most prominent reduction of the live cell population after the one-day experiment (day 1—from 84.00% to 68.12%, **** *p* < 0.0001; day 2—from 84.00% to 67.86%, ** *p* < 0.0015).
Wali et al. (2017) [76]	In vitro	BT-20, MDA-MB-231, MDA-MB-468, BT-549, MDA-MB-436, HCC38	PTX (0.03, 0.01, 0.03, 0.1, 1 µM) Incubation: 72 h	ATP-based CellTiter-Glo^®^ luminescent; Cell viability assay	PTX reduced cell viability with increased concentrations in most cell lines. MDA-MB-231 was the less sensitive cell line. The highest concentration ≤ IC50 was 3 nM.
Wang et al. (2013) [21]	In vitro	MDA-MB-231 BRCA	CAP. Plasma device: 60 V/6 A, He gas flow rate of 4.6 L/min Incubation: 24, 48, and 120 h Exposure time: 30, 60, and 90 s	MTS assay	All CAP-treated groups showed significantly inhibited cell proliferation after 3 and 5 days (*p* < 0.01).
Wang et al. (2021) [77]	In vitro	MDA-MB-231, MDA-MB-468	PAM. Plasma device: Ar jet, model kINPen 09, gas flow rate of 5.0 L/m Exposure time: 10 min PAM was diluted to different concentrations as designated by the percent remaining (e.g., 70% 10PAM refers to 70% concentration at use) Incubation: 24 h	Cell viability assay	The cell viability of MDA-MB-468 and MDA-MB-231 cells subjected to 100% PAM was 40.29 ± 6% and 16.02 ± 5.02%, respectively. Treatment did not influence the attached cell numbers of MDA-MB-468 cells but did inhibit the MDA-MB-231 cell line, which indicated MDA-MB-231 cells were more sensitive than were the other cancer lines.
Wesierska-Gadek et al. (2015) [78]	In vitro	BT-20	Olaparib (from 1 to 10 µM) Incubation: 24, 48, and 72 h	CellTiter-Glo^®^ cell viability assay (correlated with ATP levels)	There were no significant variations in the number of viable cells or increase of apoptosis.
Xiang et al. (2018) [79]	In vitro and in vivo	(1) MDA-MB-231, MDA-MB-468 (2) Female BALB/c mice; 1 × 10^6^ MDA-MB-231 cells were injected subcutaneously	PAM. Plasma device: 1.0 to 1.4 kV, 8.8 kHz, He gas flow was 1 L/min Solution: 2 mL of medium; Exposure time: 1, 2, 3, 4, and 5 min Incubation: 24 h	(1) Cell counting kit-8; Annexin V/PI (FC) (2) Tumor growth and weight	(1) Five-minute PAM reduced the viability to 0.41 and 0.46, respectively, for MDA-MB-231 and MDA-MB-468 cells. The relative apoptosis increased on both cell lines compared to healthy cells. (2) Tumor growth was inhibited, and tumor weight dropped considerably after PAM treatment (from 4.053 g to 0.787 g, *p* = 4.69 × 10^−4^). No visible side effects were observed.
Yan et al. (2015) [80]	In vitro	MDA-MB-231	PAM. Plasma device: 3.16 kV, 5 W, He gas flow rate was 4.7 L/min (1) Cell concentrations: 20,000, 40,000, and 80,000 cells/mL Exposure time: 30, 60, 90, and 120 s (2) Well number on a plate: 6, 12, 24, and 48 (3) Volume media: 1, 2, 3, and 4 mL Exposure time: 60 s Incubation: 72 h	MTT assay	(1) The anti-tumor capacity increased as the treatment time exposure increased and decreased as the cell seeding confluence decreased. (2) One minute after PAM, proliferation decreased as the size of the wells decreased. The effect of treatment was reduced 2/3 in the 48-well plate compared to the 6-well plate. (3) Relative viability significantly increased as the volume of media increased from 1 to 4 mL.
Yan et al. (2017) [81]	In vitro	MDA-MB-231	PAM. Plasma device: 3.16 kV, 30 kHz, He gas flow rate was 4.7 L/min Solution: Ringer’s solution mixed with DMEM or only medium; Exposure time: 60 s Incubation: 20 min, 1, 2, 3, and 4 h	MTT assay	Removing PAM 2, 3, or 4 h after the treatment did not change the effect of PAM on cell viability. When PAM was removed 1 h after treatment, the cytotoxicity was not as severe. The dilution remarkably impacted the anti-cancer capacity of the PAM solutions.
Yaourtis et al. (2023) [82]	In vitro	MDA-MB-231 (spindle and stellar phenotype)	DOX (Serial dilutions, from 0 to 10 µM) Incubation: 72 h	MTT assay	Spindle phenotype: IC50 = 0.31 ± 0.05 µM, *p* > 0.05 Stellar phenotype: IC50 = 0.25 ± 0.05 µM, *p* > 0.05
Zasadil et al. (2014) [83]	In vitro	MDA-MB-231; CAL51	PTX (5, 10, 20, 50, or 100 nM) Incubation: 24,72 and 120 h	Trypan blue; Clonogenic assay	Low nanomolar concentrations of PTX caused a decrease in live cell numbers over 120 h. Colony formation was substantially inhibited at concentrations ≥5 nM after 14 days.
Zhou et al. (2020) [84]	In vivo	Female BALB/c mice; 3 × 10^6^ MDA-MB-231 cells were injected subcutaneously in the right forelimbs	CAP or PAM. Plasma device: 5 kV, 8.8 kHz; He gas flow was 0.2 L/min CAP Exposure time: 5 min PAM injection treatment Solution: 2 mL of PBS Exposure time: 10 min It was subcutaneously administered into two slots of the tumor in each mouse at 100 µL/slot. Treatments were repeated every 72 h until death or the end of study (30 days).	Tumor monitoring	All mice in the control group died within 27 days, and all mice from the CAP direct group survived to the last day. The 30-day survival of mice in the CAP group was significantly higher than that of the PAM group (*p* = 4.9 × 10^−4^). Both treatments significantly inhibited tumor growth (CAP: *p* = 0.044 for CAP; PAM: *p* = 0.017). However, the growth of the tumors in the PAM group was more suppressed than that in the CAP group.

Ar—argon; ATP—adenosine triphosphate; CAB—capecitabine; CAP—cold atmospheric plasma; CAR—carboplatin; CCP—cyclophosphamide; CI—combination index; DI—deionized; DMEM—Dulbecco’s Modified Eagle Medium; DOC—docetaxel; DOX—doxorubicin; EPI—epirubicin; FC—flow cytometry; He—helium; H&E—hematoxylin and eosin; IC50—half-maximal inhibitory concentration; IC75—concentration to inhibit 75%; LDM—low-dose metronomic; MTD—maximum tolerated dose; MTS—3-(4,5-dimethylthiazol-2-yl)-5-(3-carboxymethoxyphenyl)-2-(4-sulfophenyl)-2H-tetrazolium; MTT—3-(4,5-dimethylthiazol-2-yl)-2,5-diphenyl-2H-tetrazolium bromide; N_2_—molecular nitrogen; PAM—plasma-activated media; PI—propidium iodide; PPCs—peak plasma concentrations; PTX—paclitaxel; SCID—severe combined immunodeficiency; SRB—sulforhodamine B; WST—water-soluble tetrazolium; UP—ultrapure, *—*p* < 0.05; **—*p* < 0.01; ***—*p* < 0.001; ****—*p* < 0.0001.

**Table 3:** Key aspects of this systematic review.

Key Aspect	Highlights
TNBC	(1) Due to its aggressive nature and poor prognosis compared to other molecular subtypes, TNBC requires a broader range of treatment options. (2) Chemotherapy is a well-established treatment for TNBC, but it comes with associated adverse effects. The selection of chemotherapy drugs, particularly paclitaxel, docetaxel, cyclophosphamide, doxorubicin, olaparib, carboplatin, and capecitabine, was based on ESMO and ASCO guidelines.
Plasma treatment	Cold plasma, investigated across various medical fields, including tumor therapy, shows promise in cancer treatment, including TNBC, with results indicating cell death.
Search	(1) Initial retrieval of 19,364 studies from four databases. (2) After screening, 59 articles were included in the systematic review. Articles were published between 1986 and 2023, with focus on the efficacy of chemotherapy drugs and CAP treatment.
In vitro studies	(1) In vitro studies demonstrated a reduction in cell proliferation with various chemotherapy concentrations tested, and additive interactions were observed between paclitaxel and carboplatin. (2) Different strategies, including CAP and PAM, exhibited a time-dependent reduction in TNBC cell viability, with apoptosis being the predominant type of cell death. (3) Combination therapies involving cold plasma and chemotherapy drugs tended to improve cytotoxicity in TNBC cell lines.
In vivo studies	Animal models are crucial for assessing therapy efficacy. Studies demonstrated inhibition of tumor growth with no noticeable adverse effects in animal models regarding CAP treatment.
Limitations	Heterogeneity between studies, including methodological approaches and lack of detailed information, poses limitations to meta-analysis studies and conclusive interpretations. This emphasizes the need for standardized methodologies.
Future directions	(1) This systematic review underscores the need for further research using standardized methodologies to address current limitations and advance clinical translation. (2) Studies directly comparing CAP, PAM, and standard chemotherapy regimens should be performed, including the evaluation of cell death and associated mechanisms of action. (3) Conducting studies to unravel specific protein alterations after CAP and PAM treatment might be a strategy for establishing combinations with drugs used in clinical practice. (4) Future studies should consider other animal models and explore combination therapies. Cold plasma therapy could potentially reduce chemotherapy-associated side effects by lowering drug concentrations, necessitating further investigation in this field. (5) The use of patient-derived xenografts must be considered as a key approach to verifying the effects of CAP. The findings should be correlated with patients’ clinical data.

## Data Availability

The data presented in this study are available in the manuscript.

## Supplementary Materials

The following supporting information can be downloaded at https://www.mdpi.com/article/10.3390/ijms25063254/s1.
