# Supplementary material for: Efficacy of Cold Atmospheric Plasma vs. Chemotherapy in Triple-Negative Breast Cancer: A Systematic Review
Source: Int J Mol Sci. 2024 Mar 13;25(6):3254. doi: 10.3390/ijms25063254 (PMC10970295; doi:10.3390/ijms25063254)
Supplement: Supplementary file 1 [file ijms-25-03254-s001.zip › ijms-2851679-supplementary.pdf]

Supplemental Material

1. Search strategy

1.1 PubMed

1.1.1 TNBC and Chemotherapy

("Triple Negative Breast Neoplasms"[Mesh] OR "Triple Negative Breast Neoplasm*" OR “ER-Negative PR-Negative HER2-Negative Breast Neoplasm*” OR “ER Negative PR Negative HER2 Negative Breast Neoplasm*” OR “Triple-Negative Breast Cancer*” OR “Breast Cancer, Triple-Negative” OR “Breast Cancers, Triple-Negative” OR “Triple-Negative Breast Neoplasm*” OR “Breast Neoplasm, Triple-Negative” OR “Breast Neoplasms, Triple-Negative” OR “ER-Negative PR-Negative HER2-Negative Breast Cancer*” OR “ER Negative PR Negative HER2 Negative Breast Cancer*” OR “Triple Negative Breast Cancer*” OR TNBC OR “HCC2157” OR “HCC1599” OR “HCC1937” OR “HCC1143” OR “MDA-MB-468” OR “HCC38” OR “HCC70” OR “HCC1806” OR “HCC1187” OR “DU4475” OR “BT-549” OR “Hs 578T” OR “MDA-MB-231” OR “MDA-MDB-231” OR “MDA-MB-436” OR “MDA-MB-157” OR “MDA-MB-453” OR “BT-20” OR “HCC1395”) AND ("Doxorubicin"[Mesh] OR “Doxorubicin*” OR “Farmiblastina” OR “Ribodoxo” OR “Rubex” OR “Adriamycin” OR “Adriblastin*” OR “Adriablastin*” OR “Adrimedac” OR “DOXO-cell” OR “DOXO cell” OR “Doxolem” OR “Doxotec” OR “Myocet” OR “Onkodox” OR "Epirubicin"[Mesh] OR “Epirubicin” OR “4'-Epidoxorubicin” OR “4' Epidoxorubicin” OR “4'-Epi-Doxorubicin” OR “4' Epi Doxorubicin” OR “4'-Epi-Adriamycin” OR “4' Epi Adriamycin” OR “4'-Epiadriamycin” OR “4' Epiadriamycin” OR “4'-Epi-DXR” OR “4' Epi DXR” OR “EPI-cell” OR “EPI cell” OR “EPIcell” OR “Epilem” OR “Farmorubicin*” OR “IMI-28” OR “IMI 28” OR “IMI28” OR “NSC-256942” OR “NSC 256942” OR “NSC256942” OR “Ellence” OR “Pharmorubicin” OR "Cyclophosphamide"[Mesh] OR “Cyclophosphamide” OR “Sendoxan” OR “B-518” OR “B 518” OR “B518” OR “Cytophosphan*” OR “Cytoxan” OR “Endoxan” OR “Neosar” OR “NSC-26271” OR “NSC 26271” OR “NSC26271” OR “Procytox” OR “(+,-)-2-(bis(2-Chloroethyl)amino)tetrahydro-2H-1,3,2-oxazaphosphorine 2-Oxide Monohydrate” OR “Cyclophosphane” OR "Paclitaxel"[Mesh] OR “Paclitaxel” OR “Anzatax” OR “NSC-125973” OR “NSC 125973” OR “NSC125973” OR “Taxol” OR “Paxene” OR “Praxel” OR “7-epi-Taxol” OR “Onxol” OR "Docetaxel"[Mesh] OR “Docetaxel” OR “Docetaxol” OR “Taxoltere Metro” OR “RP 56976” OR “RP-56976” OR “RP56976” OR “Taxotere” OR “N-Debenzoyl-N-tert-butoxycarbonyl-10-deacetyltaxol” OR “N Debenzoyl N tert butoxycarbonyl 10 deacetyltaxol” OR “NSC 628503” OR "Carboplatin"[Mesh] OR “Carboplat*” OR “cis-Diammine(cyclobutanedicarboxylato)platinum II” OR “CBDCA” OR “Paraplatin*” OR “Platinwas” OR “Ribocarbo” OR “Neocarbo” OR “Carbosin” OR “Carbotec” OR “Ercar” OR “JM-8” OR “JM 8” OR “JM8” OR “Nealorin” OR “NSC-241240” OR “NSC 241240” OR “NSC241240” OR “Blastocarb” OR "Capecitabine"[Mesh] OR “Capecitabine” OR “N(4)-pentyloxycarbonyl-5'-deoxy-5-fluorocytidine” OR “Xeloda” OR "olaparib" [Supplementary Concept] OR olaparib OR “AZD 2281” OR “AZD2281” OR “AZD-2281” OR “AZD221” OR “Lynparza”), using language filters, including articles written in Portuguese, English, Spanish and French.

1.1.2 TNBC and Cold Plasma

("Triple Negative Breast Neoplasms"[Mesh] OR "Triple Negative Breast Neoplasm*" OR “ER-Negative PR-Negative HER2-Negative Breast Neoplasm*” OR “ER Negative PR Negative HER2 Negative Breast Neoplasm*” OR “Triple-Negative Breast Cancer*” OR “Breast Cancer, Triple-Negative” OR “Breast Cancers, Triple-Negative” OR “Triple-Negative Breast Neoplasm*” OR “Breast Neoplasm, Triple-Negative” OR “Breast Neoplasms, Triple-Negative” OR “ER-Negative PR-Negative HER2-Negative Breast Cancer*” OR “ER Negative PR Negative HER2 Negative Breast Cancer*” OR “Triple Negative Breast Cancer*” OR TNBC OR “HCC2157” OR “HCC1599” OR “HCC1937” OR “HCC1143” OR “MDA-MB-468” OR “HCC38” OR “HCC70” OR “HCC1806” OR “HCC1187” OR “DU4475” OR “BT-549” OR “Hs 578T” OR “MDA-MB-231” OR “MDA-MDB-231” OR “MDA-MB-436” OR “MDA-MB-157” OR “MDA-MB-453” OR “BT-20” OR “HCC1395”) AND ("Plasma Gases"[Mesh] OR "Plasma Gases" OR “Gases, Plasma” OR “Cold Plasma” OR “Plasma, Cold” OR “Non-Thermal Atmospheric Pressure Plasma” OR “Non Thermal Atmospheric Pressure Plasma” OR “Thermal Plasma” OR “Plasma, Thermal” OR CAP), using language filters, including articles written in Portuguese, English, Spanish and French.

1.2 Web of Science

1.2.1 TNBC and Chemotherapy

("Triple Negative Breast Neoplasm*" OR “ER-Negative PR-Negative HER2-Negative Breast Neoplasm*” OR “ER Negative PR Negative HER2 Negative Breast Neoplasm*” OR “Triple-Negative Breast Cancer*” OR

“Breast Cancer*, Triple-Negative” OR “Triple-Negative Breast Neoplasm*” OR “Breast Neoplasm*, Triple-Negative” OR “ER-Negative PR-Negative HER2-Negative Breast Cancer*” OR “ER Negative PR Negative HER2 Negative Breast Cancer*” OR “Triple Negative Breast Cancer*” OR TNBC OR “HCC2157” OR “HCC1599” OR “HCC1937” OR “HCC1143” OR “MDA-MB-468” OR “HCC38” OR “HCC70” OR “HCC1806” OR “HCC1187” OR “DU4475” OR “BT-549” OR “Hs 578T” OR “MDA-MB-231” OR “MDA-MDB-231” OR “MDA-MB-436” OR “MDA-MB-157” OR “MDA-MB-453” OR “BT-20” OR “HCC1395”) AND (“Doxorubicin*” OR “Farmiblastina” OR “Ribodoxo” OR “Rubex” OR “Adriamycin” OR “Adriblastin*” OR “Adriablastin*” OR “Adrimedac” OR “DOXO-cell” OR “DOXO cell” OR “Doxolem” OR “Doxotec” OR “Myocet” OR “Onkodox” OR “Epirubicin” OR “4'-Epidoxorubicin” OR “4' Epidoxorubicin” OR “4'-Epi-Doxorubicin” OR “4' Epi Doxorubicin” OR “4'-Epi-Adriamycin” OR “4' Epi Adriamycin” OR “4'-Epiadriamycin” OR “4' Epiadriamycin” OR “4'-Epi-DXR” OR “4' Epi DXR” OR “EPI-cell” OR “EPI cell” OR “EPIcell” OR “Epilem” OR “Farmorubicin*” OR “IMI-28” OR “IMI 28” OR “IMI28” OR “NSC-256942” OR “NSC 256942” OR “NSC256942” OR “Ellence” OR “Pharmorubicin” OR “Cyclophosphamide” OR “Sendoxan” OR “B-518” OR “B 518” OR “B518” OR “Cytophosphan*” OR “Cytoxan” OR “Endoxan” OR “Neosar” OR “NSC-26271” OR “NSC 26271” OR “NSC26271” OR “Procytox” OR “(+,-)-2-(bis(2-Chloroethyl)amino)tetrahydro-2H-1,3,2-oxazaphosphorine 2-Oxide Monohydrate” OR “Cyclophosphane” OR “Paclitaxel” OR “Anzatax” OR “NSC-125973” OR “NSC 125973” OR “NSC125973” OR “Taxol” OR “Paxene” OR “Praxel” OR “7-epi-Taxol” OR “Onxol” OR “Docetaxel” OR “Docetaxol” OR “Taxoltere Metro” OR “RP 56976” OR “RP-56976” OR “RP56976” OR “Taxotere” OR “N-Debenzoyl-N-tert-butoxycarbonyl-10-deacetyltaxol” OR “N Debenzoyl N tert butoxycarbonyl 10 deacetyltaxol” OR “NSC 628503” OR “Carboplat*” OR “cis-Diammine(cyclobutanedicarboxylato)platinum II” OR “CBDCA” OR “Paraplatin*” OR “Platinwas” OR “Ribocarbo” OR “Neocarbo” OR “Carbosin” OR “Carbotec” OR “Ercar” OR “JM-8” OR “JM 8” OR “JM8” OR “Nealorin” OR “NSC-241240” OR “NSC 241240” OR “NSC241240” OR “Blastocarb” OR “Capecitabine” OR “N(4)-pentyloxycarbonyl-5'-deoxy-5-fluorocytidine” OR “Xeloda” OR olaparib OR “AZD 2281” OR “AZD2281” OR “AZD-2281” OR “AZD221” OR “Lynparza”), using language filters, including articles written in Portuguese, English and Spanish.

1.2.2 TNBC and Cold Plasma

("Triple Negative Breast Neoplasm*" OR “ER-Negative PR-Negative HER2-Negative Breast Neoplasm*” OR “ER Negative PR Negative HER2 Negative Breast Neoplasm*” OR “Triple-Negative Breast Cancer*” OR “Breast Cancer*, Triple-Negative” OR “Triple-Negative Breast Neoplasm*” OR “Breast Neoplasm*, Triple-Negative” OR “ER-Negative PR-Negative HER2-Negative Breast Cancer*” OR “ER Negative PR Negative HER2 Negative Breast Cancer*” OR “Triple Negative Breast Cancer*” OR TNBC OR “HCC2157” OR “HCC1599” OR “HCC1937” OR “HCC1143” OR “MDA-MB-468” OR “HCC38” OR “HCC70” OR “HCC1806” OR “HCC1187” OR “DU4475” OR “BT-549” OR “Hs 578T” OR “MDA-MB-231” OR “MDA-MDB-231” OR “MDA-MB-436” OR “MDA-MB-157” OR “MDA-MB-453” OR “BT-20” OR “HCC1395”) AND ("Plasma Gases" OR “Gases, Plasma” OR “Cold Plasma” OR “Plasma, Cold” OR “Non-Thermal Atmospheric Pressure Plasma” OR “Non Thermal Atmospheric Pressure Plasma” OR “Thermal Plasma” OR “Plasma, Thermal” OR CAP), using language filters, including articles written in Portuguese, English and Spanish.

1.3 Embase

1.3.1 TNBC and Chemotherapy

('triple negative breast cancer'/exp OR 'triple negative breast neoplasm*':ti,ab,kw OR 'er-negative pr-negative her2-negative breast neoplasm*':ti,ab,kw OR 'er negative pr negative her2 negative breast neoplasm*':ti,ab,kw OR 'triple-negative breast cancer*':ti,ab,kw OR 'breast cancer, triple-negative':ti,ab,kw OR 'breast cancers, triple-negative':ti,ab,kw OR 'triple-negative breast neoplasm*':ti,ab,kw OR 'breast neoplasm, triple-negative':ti,ab,kw OR 'breast neoplasms, triple-negative':ti,ab,kw OR 'er-negative pr-negative her2-negative breast cancer*':ti,ab,kw OR 'er negative pr negative her2 negative breast cancer*':ti,ab,kw OR 'triple negative breast cancer*':ti,ab,kw OR tnbc:ti,ab,kw OR hcc2157:ti,ab,kw OR hcc1599:ti,ab,kw OR hcc1937:ti,ab,kw OR 'hcc1143 cell line'/exp OR hcc1143:ti,ab,kw OR 'mda-mb-468 cell line'/exp OR 'mda mb 468':ti,ab,kw OR 'hcc38 cell line'/exp OR hcc38:ti,ab,kw OR 'hcc70 cell line'/exp OR hcc70:ti,ab,kw OR 'hcc1806 cell line'/exp OR hcc1806:ti,ab,kw OR 'hcc1187 cell line'/exp OR hcc1187:ti,ab,kw OR 'du4475 cell line'/exp OR du4475:ti,ab,kw OR 'bt-549 cell line'/exp OR 'bt 549':ti,ab,kw OR 'hs 578t cell line'/exp OR 'hs 578t':ti,ab,kw OR 'mda-mb-231 cell line'/exp OR 'mda mb 231':ti,ab,kw OR 'mda mdb 231':ti,ab,kw OR 'mda-mb-436 cell line'/exp OR 'mda mb 436':ti,ab,kw OR 'mda-mb-157 cell line'/exp OR 'mda mb 157':ti,ab,kw OR 'mda-mb-453 cell line'/exp OR 'mda mb 453':ti,ab,kw OR 'bt-20 cell line'/exp OR 'bt 20':ti,ab,kw OR 'hcc1395 cell line'/exp OR hcc1395:ti,ab,kw) AND ('doxorubicin'/exp OR doxorubicin*:ti,ab,kw OR farmiblastina:ti,ab,kw OR ribodoxo:ti,ab,kw OR rubex:ti,ab,kw OR adriamycin:ti,ab,kw OR adriblastin*:ti,ab,kw OR adriablastin*:ti,ab,kw OR adrimedac:ti,ab,kw OR 'doxo cell':ti,ab,kw OR doxolem:ti,ab,kw OR doxotec:ti,ab,kw OR myocet:ti,ab,kw OR onkodox:ti,ab,kw OR

'epirubicin'/exp OR epirubicin:ti,ab,kw OR '4 epidoxorubicin':ti,ab,kw OR '4 epi doxorubicin':ti,ab,kw OR '4 epi adriamycin':ti,ab,kw OR '4 epiadriamycin':ti,ab,kw OR '4 epi dxr':ti,ab,kw OR 'epi cell':ti,ab,kw OR epicell:ti,ab,kw OR epilem:ti,ab,kw OR farmorubicin*:ti,ab,kw OR 'imi 28':ti,ab,kw OR 'imi28':ti,ab,kw OR 'nsc 256942':ti,ab,kw OR nsc256942:ti,ab,kw OR ellence:ti,ab,kw OR pharmorubicin:ti,ab,kw OR 'cyclophosphamide'/exp OR cyclophosphamide:ti,ab,kw OR sendoxan:ti,ab,kw OR 'b 518':ti,ab,kw OR b518:ti,ab,kw OR cytophosphan*:ti,ab,kw OR cytoxan:ti,ab,kw OR endoxan:ti,ab,kw OR neosar:ti,ab,kw OR 'nsc 26271':ti,ab,kw OR nsc26271:ti,ab,kw OR procytox:ti,ab,kw OR (+,-:ti,ab,kw AND -2-:ti,ab,kw AND bis:ti,ab,kw AND '2 chloroethyl':ti,ab,kw AND amino:ti,ab,kw AND 'tetrahydro-2h-1,3,2-oxazaphosphorine 2-oxide monohydrate':ti,ab,kw) OR cyclophosphane:ti,ab,kw OR 'paclitaxel'/exp OR paclitaxel:ti,ab,kw OR anzatax:ti,ab,kw OR 'nsc 125973':ti,ab,kw OR nsc125973:ti,ab,kw OR taxol:ti,ab,kw OR paxene:ti,ab,kw OR praxel:ti,ab,kw OR '7 epi taxol':ti,ab,kw OR onxol:ti,ab,kw OR 'docetaxel'/exp OR docetaxel:ti,ab,kw OR docetaxol:ti,ab,kw OR 'taxoltere metro':ti,ab,kw OR 'rp 56976':ti,ab,kw OR rp56976:ti,ab,kw OR taxotere:ti,ab,kw OR 'n debenzoyl n tert butoxycarbonyl 10 deacetyltaxol':ti,ab,kw OR 'nsc 628503':ti,ab,kw OR 'carboplatin'/exp OR carboplat*:ti,ab,kw OR ('cis diammine':ti,ab,kw AND cyclobutanedicarboxylato:ti,ab,kw AND 'platinum ii':ti,ab,kw) OR cbdca:ti,ab,kw OR paraplatin*:ti,ab,kw OR platinwas:ti,ab,kw OR ribocarbo:ti,ab,kw OR neocarbo:ti,ab,kw OR carbosin:ti,ab,kw OR carbotec:ti,ab,kw OR ercar:ti,ab,kw OR 'jm 8':ti,ab,kw OR jm8:ti,ab,kw OR nealorin:ti,ab,kw OR 'nsc 241240':ti,ab,kw OR nsc241240:ti,ab,kw OR blastocarb:ti,ab,kw OR 'capecitabine'/exp OR capecitabine:ti,ab,kw OR (n:ti,ab,kw AND 4:ti,ab,kw AND 'pentyloxycarbonyl 5 deoxy 5 fluorocytidine':ti,ab,kw) OR xeloda:ti,ab,kw OR 'olaparib'/exp OR olaparib:ti,ab,kw OR azd2281:ti,ab,kw OR 'azd 2281':ti,ab,kw OR azd221:ti,ab,kw OR lynparza:ti,ab,kw) AND ([english]/lim OR [french]/lim OR [portuguese]/lim OR [spanish]/lim) AND ([article]/lim OR [article in press]/lim OR [data papers]/lim OR [letter]/lim) AND ('ab initio calculation'/de OR 'animal cell'/de OR 'animal cell culture'/de OR 'animal experiment'/de OR 'animal model'/de OR 'animal tissue'/de OR 'antineoplastic protocol'/de OR 'biological model'/de OR 'bone marrow culture'/de OR 'cancer cell culture'/de OR 'cell culture'/de OR 'cell culture technique'/de OR 'chemical model'/de OR 'coculture'/de OR 'comparative effectiveness'/de OR 'comparative study'/de OR 'control group'/de OR 'controlled study'/de OR 'disease model'/de OR 'disease simulation'/de OR 'dosage schedule comparison'/de OR 'drug dosage form comparison'/de OR 'drug dose comparison'/de OR 'ex vivo study'/de OR 'experimental design'/de OR 'experimental model'/de OR 'experimental study'/de OR 'exploratory research'/de OR 'fibroblast culture'/de OR 'genetic model'/de OR 'heart cell culture'/de OR 'human'/de OR 'human cell'/de OR 'human cell culture'/de OR 'human experiment'/de OR 'human tissue'/de OR 'hybridoma cell culture'/de OR 'in vitro study'/de OR 'in vivo study'/de OR 'leukocyte culture'/de OR 'lymphocyte culture'/de OR 'mammal cell'/de OR 'mixed cell culture'/de OR 'model'/de OR 'molecular model'/de OR 'monolayer culture'/de OR 'mouse model'/de OR 'murine model'/de OR 'nerve cell culture'/de OR 'nonhuman'/de OR 'normal human'/de OR 'pilot study'/de OR 'preclinical study'/de OR 'primary cell culture'/de OR 'prospective study'/de OR 'suspension cell culture'/de OR 'three dimensional cell culture'/de OR 'tissue culture'/de OR 'tumor cell culture'/de OR 'tumor model'/de OR 'tumor spheroid'/de OR 'two dimensional cell culture'/de OR 'validation study'/de)

1.3.2 TNBC and Cold Plasma

(('triple negative breast cancer'/exp OR 'triple negative breast neoplasm*':ti,ab,kw OR 'er negative pr-negative her2-negative breast neoplasm*':ti,ab,kw OR 'er negative pr negative her2 negative breast neoplasm*':ti,ab,kw OR 'triple-negative breast cancer*':ti,ab,kw OR 'breast cancer, triple-negative':ti,ab,kw OR 'breast cancers, triplenegative': ti,ab,kw OR 'triple-negative breast neoplasm*':ti,ab,kw OR 'breast neoplasm, triple-negative':ti,ab,kw OR 'breast neoplasms, triple-negative':ti,ab,kw OR 'er-negative pr-negative her2-negative breast cancer*':ti,ab,kw OR 'er negative pr negative her2 negative breast cancer*':ti,ab,kw OR 'triple negative breast cancer*':ti,ab,kw OR tnbc:ti,ab,kw OR hcc2157:ti,ab,kw OR hcc1599:ti,ab,kw OR hcc1937:ti,ab,kw OR 'hcc1143 cell line'/exp OR hcc1143:ti,ab,kw OR 'mda-mb-468 cell line'/exp OR 'mda mb 468':ti,ab,kw OR 'hcc38 cell line'/exp OR hcc38:ti,ab,kw OR 'hcc70 cell line'/exp OR hcc70:ti,ab,kw OR 'hcc1806 cell line'/exp OR hcc1806:ti,ab,kw OR 'hcc1187 cell line'/exp OR hcc1187:ti,ab,kw OR 'du4475 cell line'/exp OR du4475:ti,ab,kw OR 'bt-549 cell line'/exp OR 'bt 549':ti,ab,kw OR 'hs 578t cell line'/exp OR 'hs 578t':ti,ab,kw OR 'mda-mb-231 cell line'/exp OR 'mda mb 231':ti,ab,kw OR 'mda mdb 231':ti,ab,kw OR 'mda-mb-436 cell line'/exp OR 'mda mb 436':ti,ab,kw OR 'mda-mb-157 cell line'/exp OR 'mda mb 157':ti,ab,kw OR 'mda-mb-453 cell line'/exp OR 'mda mb 453':ti,ab,kw OR 'bt- 20 cell line'/exp OR 'bt 20':ti,ab,kw OR 'hcc1395 cell line'/exp OR hcc1395:ti,ab,kw) AND ('plasma gas'/exp OR 'plasma gas*':ti,ab,kw OR 'gases, plasma':ti,ab,kw OR 'cold plasma'/exp OR 'cold plasma':ti,ab,kw OR 'plasma, cold':ti,ab,kw OR 'non thermal atmospheric pressure plasma'/exp OR 'non-thermal atmospheric pressure plasma':ti,ab,kw OR 'non thermal atmospheric pressure plasma':ti,ab,kw OR 'thermal plasma':ti,ab,kw OR 'plasma, thermal':ti,ab,kw OR cap:ti,ab,kw) AND ([english]/lim OR [french]/lim OR [portuguese]/lim OR [spanish]/lim)) AND ('article'/it OR 'note'/it) AND ('animal cell'/de OR 'animal experiment'/de OR 'animal model'/de OR 'animal tissue'/de OR 'cancer cell culture'/de OR 'cancer model'/de OR 'cell culture'/de OR 'comparative study'/de OR 'controlled study'/de OR 'drug dosage form

comparison'/de OR 'human'/de OR 'human cell'/de OR 'human tissue'/de OR 'in vitro study'/de OR 'in vivo study'/de OR 'mouse model'/de OR 'nonhuman'/de OR 'pilot study'/de OR 'prospective study'/de OR 'tumor spheroid'/de OR 'two dimensional cell culture'/de)

1.4 Cochrane Library

1.4.1 TNBC and Chemotherapy

#1 MeSH descriptor: [Triple Negative Breast Neoplasms] explode all trees 431
#2 "Triple Negative Breast Neoplasm" 3
#3 "Triple Negative Breast Neoplasms" 437
#4 "ER-Negative PR-Negative HER2-Negative Breast Neoplasm" 0
#5 "ER-Negative PR-Negative HER2-Negative Breast Neoplasms" 0
#6 "Triple-Negative Breast Cancer" 1565
#7 "Triple-Negative Breast Cancers" 56
#8 "Breast Cancer, Triple-Negative" 17
#9 "Breast Cancers, Triple-Negative" 1
#10 "Triple-Negative Breast Neoplasm" 3
#11 "Triple-Negative Breast Neoplasms" 437
#12 "Breast Neoplasm, Triple-Negative" 0
#13 "Breast Neoplasms, Triple-Negative" 24
#14 "ER-Negative PR-Negative HER2-Negative Breast Cancer" 0
#15 "ER-Negative PR-Negative HER2-Negative Breast Cancers" 0
#16 TNBC 1129
#17 HCC2157 0
#18 HCC1599 0
#19 HCC1937 0
#20 HCC1143 0
#21 MDA-MB-468 7
#22 HCC38 2
#23 HCC70 1
#24 HCC1806 0
#25 HCC1187 1
#26 DU4475 0
#27 BT-549 3
#28 "Hs 578T" 0
#29 MDA-MB-231 35
#30 MDA-MDB-231 0
#31 MDA-MB-436 3
#32 MDA-MB-157 1
#33 MDA-MB-453 7
#34 BT-20 9
#35 HCC1395 0
#36 #1 OR #2 OR #3 OR #4 OR #5 OR #6 OR #7 OR #8 OR #9 OR #10 OR #11 OR #12 OR #13 OR #14 OR #15 OR #16 OR #17 OR #18 OR #19 OR #20 OR #21 OR #22 OR #23 OR #24 OR #25 OR #26 OR #27 OR #28 OR #29 OR #30 OR #31 OR #32 OR #33 OR #34 OR #35 1722
#37 MeSH descriptor: [Doxorubicin] explode all trees 5444
#38 Doxorubicin* 8635
#39 Farmiblastina 5
#40 Ribodoxo 7
#41 Rubex 7
#42 Adriamycin 1941
#43 Adriblastin* 32
#44 Adriablastin* 22
#45 Adrimedac 5
#46 DOXO-cell 8
#47 Doxolem 6
#48 Doxotec 4
#49 Myocet 80
#50 Onkodox 5
#51 MeSH descriptor: [Epirubicin] explode all trees 1343
#52 Epirubicin 3452
#53 "4'-Epidoxorubicin" 48
#54 "4'-Epi-Doxorubicin" 23
#55 "4'-Epi-Adriamycin" 13
#56 "4'-Epiadriamycin" 17
#57 "4'-Epi-DXR" 8

#58	EPI-cell	5
#59	EPIcell	5
#60	Epilem	3
#61	Farmorubicin*	46
#62	IMI-28	12
#63	IMI28	10
#64	NSC-256942	9
#65	NSC256942	8
#66	Ellence	10
#67	Pharmorubicin	12
#68	MeSH descriptor: [Cyclophosphamide] explode all trees	6127
#69	Cyclophosphamide	13392
#70	Sendoxan	19
#71	B-518	8
#72	B518	4
#73	Cytophosphan*	7
#74	Cytoxan	206
#75	Endoxan	118
#76	Neosar	16
#77	NSC-26271	24
#78	NSC26271	3
#79	Procytox	7
#80	"(+,-)-2-(bis(2-Chloroethyl)amino)tetrahydro-2H-1,3,2-oxazaphosphorine 2-Oxide Monohydrate"	0
#81	Cyclophosphane	8
#82	MeSH descriptor: [Paclitaxel] explode all trees	4445
#83	Paclitaxel	12029
#84	Anzatax	10
#85	NSC-125973	9
#86	NSC125973	2
#87	Taxol	569
#88	Paxene	8
#89	Praxel	6
#90	"7-epi-Taxol"	2
#91	Onxol	5
#92	MeSH descriptor: [Docetaxel] explode all trees	2607
#93	Docetaxel	8148
#94	Docetaxol	19
#95	"Taxoltere Metro"	0
#96	RP-56976	7
#97	RP56976	2
#98	Taxotere	532
#99	"N-Debenzoyl-N-tert-butoxycarbonyl-10-deacetyltaxol"	0
#100	"NSC 628503"	5
#101	MeSH descriptor: [Carboplatin] explode all trees	2918
#102	Carboplat*	8410
#103	"cis-Diammine(cyclobutanedicarboxylato)platinum II"	6
#104	CBDCA	265
#105	Paraplatin*	62
#106	Platinwas	10
#107	Ribocarbo	13
#108	Neocarbo	10
#109	Carbosin	11
#110	Carbotec	10
#111	Ercar	10
#112	JM-8	15
#113	JM8	19
#114	Nealorin	10
#115	NSC-241240	13
#116	NSC241240	8
#117	Blastocarb	10
#118	MeSH descriptor: [Capecitabine] explode all trees	1564
#119	Capecitabine	4593
#120	"N(4)-pentyloxycarbonyl-5'-deoxy-5-fluorocytidine"	0
#121	Xeloda	370
#122	olaparib	821
#123	AZD-2281	6

#124 AZD2281 75
#125 AZD221 0
#126 Lynparza 58
#127 #37 OR #38 OR #39 OR #40 OR #41 OR #42 OR #43 OR #44 OR #45 OR #46 OR #47 OR #48 OR #49 OR #50 OR #51 OR #52 OR #53 OR #54 OR #55 OR #56 OR #57 OR #58 OR #59 OR #60 OR #61 OR #62 OR #63 OR #64 OR #65 OR #66 OR #67 OR #68 OR #69 OR #70 OR #71 OR #72 OR #73 OR #74 OR #75 OR #76 OR #77 OR #78 OR #79 OR #80 OR #81 OR #82 OR #83 OR #84 OR #85 OR #86 OR #87 OR #88 OR #89 OR #90 OR #91 OR #92 OR #93 OR #94 OR #95 OR #96 OR #97 OR #98 OR #99 OR #100 OR #101 OR #102 OR #103 OR #104 OR #105 OR #106 OR #107 OR #108 OR #109 OR #110 OR #111 OR #112 OR #113 OR #114 OR #115 OR #116 OR #117 OR #118 OR #119 OR #120 OR #121 OR #122 OR #123 OR #124 OR #125 OR #126 42179
#128 #36 AND #127 1121

1.4.2 TNBC and Cold Plasma

#1 MeSH descriptor: [Triple Negative Breast Neoplasms] explode all trees 431
#2 "Triple Negative Breast Neoplasm" 3
#3 "Triple Negative Breast Neoplasms" 437
#4 "ER-Negative PR-Negative HER2-Negative Breast Neoplasm"0
#5 "ER-Negative PR-Negative HER2-Negative Breast Neoplasms" 0
#6 "Triple-Negative Breast Cancer" 1565
#7 "Triple-Negative Breast Cancers" 56
#8 "Breast Cancer, Triple-Negative" 17
#9 "Breast Cancers, Triple-Negative" 1
#10 "Triple-Negative Breast Neoplasm" 3
#11 "Triple-Negative Breast Neoplasms" 437
#12 "Breast Neoplasm, Triple-Negative"0
#13 "Breast Neoplasms, Triple-Negative" 24
#14 "ER-Negative PR-Negative HER2-Negative Breast Cancer" 0
#15 "ER-Negative PR-Negative HER2-Negative Breast Cancers" 0
#16 TNBC 1129
#17 HCC2157 0
#18 HCC1599 0
#19 HCC1937 0
#20 HCC1143 0
#21 MDA-MB-468 7
#22 HCC38 2
#23 HCC70 1
#24 HCC1806 0
#25 HCC1187 1
#26 DU4475 0
#27 BT-549 3
#28 "Hs 578T" 0
#29 MDA-MB-231 35
#30 MDA-MDB-231 0
#31 MDA-MB-436 3
#32 MDA-MB-157 1
#33 MDA-MB-453 7
#34 BT-20 9
#35 HCC1395 0
#36 #1 OR #2 OR #3 OR #4 OR #5 OR #6 OR #7 OR #8 OR #9 OR #10 OR #11 OR #12 OR #13 OR #14 OR #15 OR #16 OR #17 OR #18 OR #19 OR #20 OR #21 OR #22 OR #23 OR #24 OR #25 OR #26 OR #27 OR #28 OR #29 OR #30 OR #31 OR #32 OR #33 OR #34 OR #35 1722
#37 MeSH descriptor: [Plasma Gases] explode all trees 45
#38 "Plasma Gases" 46
#39 "Gases, Plasma" 11
#40 "Cold Plasma" 23
#41 "Plasma, Cold" 2
#42 "Non-Thermal Atmospheric Pressure Plasma" 5
#43 "Non Thermal Atmospheric Pressure Plasma" 5
#44 "Thermal Plasma" 6
#45 "Plasma, Thermal" 0
#46 CAP 5857
#47 #38 OR #39 OR #40 OR #41 OR #42 OR #43 OR #44 OR #45 OR #465923
#48 #36 AND #47

2. Results

Table S1 – Quantitative summary of cell viability/proliferation reduction outcomes.

ARTICLE (Year)	CELL LINE	STUDY	TREATMENT	DOSE	PERIOD OF INCUBATION	MEAN (reduction) ± SD (if applicable)
Xiang et al (2018)	MDA-MB-231	In vivo	PAM treatment	5 min	24 h	19.4%
Arun et al (2016)	MDA-MB-231	MTT assay	Doxorubicin	1 μM	12h	1,0%
				6 μM		20,0%
				1 μM	24h	35,0%
				2 μM		35,0%
Chen et al (2017)	MDA-MB-231	MTT assay	CAP treatment	Air	24h	27.4%
				Helium	24h	14.7%
				Air	48h	73.1%
				Helium	48h	22.8%
				Nitrogen	48h	14.1%
				Water	48h	13.5%
Mihai et al (2022)	MDA-MB-231	MTT assay	Paclitaxel	0.001μM	24h	105,0%
				0.01 μM		63.05%
				0.001μM	48h	92.79%
				0.01 μM		28.31%
			PAM treatment	30 and 60s	48h	75,0%
			Paclitaxel + PAM treatment	Several combinations	24h	Between 66.23% and 63.72%
					48h	Between 81.20% and 80.05%
		Area (spheroids)	PAM treatment	15 s	24h	23.83% vs control and 20.95% vs PTX
Subramanian et al (2020)	MDA-MB-231	MTT assay	PAM treatment	6 min	24h	19,0%
				12 min		45,0%
				18 min		76,0%
				Acid		7,0%

				60 µL		3,0%
				80 µL		2,0%
				100 µL		3,0%
				150 µL		34,0%
				200 µL		80,0%
				6 min		19,0%
				12 min		37,0%
				12 min		21,0%
				18 min		74,0%
				18 min		61,0%
Chuang et al (2012)	MDA-MB-231	MTT assay	Olaparib	>100 µM	72h	50,0%
	MDA-MB-468			18 µM		
	Cal5			9.5 µM		
Izbicka et al (2005)	MDA-MB-231	MTT assay	Docetaxel	0.1 nM	72h	25,0%
				0.5 nM		50,0%
				5 nM		75,0%
			Paclitaxel	0.1nM		25,0%
				1 nM		50,0%
				5 nM		75,0%
Kim et al (2003)	MDA-MB-231	MTT assay	Doxorubicin	0.3 µM	48h	50,0%
			Paclitaxel	0.03 µM		
Moschetta-Pinheiro et al (2022)	MDA-MB-468	MTT assay	Carboplatin	10 µM	24h	50,0%
Parrella et al (2014)	MDA-MB-231	MTT assay	Doxorubicin	19 µM	24h	50,0%
				4 µM	72h	
			Capecitabine	5150 µM	24h	
				2790 µM	72h	
Pilco-Ferreto & Calaf (2016)	MDA-MB-231	MTT assay	Doxorubicin	1 µM	48h	50,0%

Taherian et al (2012)	MDA-MB-231	MTT assay	Doxorubicin	887.75 µM	48h	50,0%
			Docetaxel	634.58 µM		
Tassone et al (2003)	MDA-MB-231	MTT assay	Doxorubicin	5-10 µM	48h	50,0%
			Paclitaxel	0.01-0.02 µM		
	HCC1937		Doxorubicin	45-50 µM		
			Paclitaxel	2 µM		
Yaourtis et al (2023)	MDA-MB-231 (Spindle and stellar phenotype)	MTT assay	Doxorubicin	0.31±0.05µM	72h	50,0%
				0.25±0.05µM		50,0%
Frankfurt & Krishan (2003)	MDA-MB-468	MTT assay	Doxorubicin	0.05 µM	48h	50,0%
			Paclitaxel	0.01 µM		
		SRB assay	Doxorubicin	0.1 µM		
			Paclitaxel	0.01 µM		
Blois et al (2011)	MDA-MB-231	SRB assay	Paclitaxel	0.07 nM	48h	50,0%
Norris et al (2013)	HCC-1937	SRB assay	Olaparib	100 nM	120h	50,0%
Oncul et al (2017)	MDA-MB-231	SRB assay	Doxorubicin	50 nM	48h	111.64±14.59%
				100 nM		95.25±3.14%
				200 nM		92.68±5.80%
				400 nM		89.02±6.32%
				800 nM		75.94±4.85%
				1000 nM		84.04±9.74%
				1500 nM		82.32±8.71%
				2000 nM		76.94±4.37%
				3000 nM		74.597±4.64%
				4000 nM		68.90±4.01%
				8000 nM		41.11±4.73%
Chuang et al (2012)	MDA-MB-231	Clonogenic assay	Olaparib	4.5 µM	72h	50,0%
	MDA-MB-468			0.2 µM		

	Cal51			0.4 µM		
Hernández-Vargas et al (2007)	MDA-MB-231	Clonogenic assay	Docetaxel	2 nM	24h	75,0%
Lafontaine et al (2020)	BT549	Clonogenic assay	CAP treatment	7.8 s	6 days	50,0%
	Hs578T			18.4 s		
	MDA-MB-157			4.6 s		
	MDA-MB-231			1.6 s		
	MDA-MB-468			6 s		
Morse et al (2005)	MDA-MB-231	Clonogenic assay	Docetaxel	10nmol/L	24h	50,0%
				10nmol/L	48h	
				10nmol/L	72h	
Almeida-Ferreira et al (2022)	HCC1806	Annexin V/PI (FC)	CAP treatment	60 s	24h	80.5±1.59% to 64.67±2.16%
				120s	24h	80.5±1.59% to 65.00±3.39%
Morse et al (2005)	MDA-MB-231	Annexin V/PI (FC)	Docetaxel	10nmol/L	24h	13.4±25.6%
				100nmol/L		51.5±31.6%
				10nmol/L	48h	21.8±2.9%
				100nmol/L		62.5±3.1%
				10nmol/L	72h	12.9±2.7%
				100nmol/L		68.4±2.8%
Stope et al (2020)	MDA-MB-231	Annexin V/PI (FC)	CAP tretament	60s	4h-120h	4.5%
			PAM treatment	60s	4h-120h	3.0%
Wang et al (2021)	MDA-MB-231	Annexin V/PI (FC)	PAM treatment	10 min	24h	83.98±5.02%
	MDA-MB-468		PAM treatment	10 min	24h	59.71±6%
Xiang et al (2018)	MDA-MB-231	Annexin V/PI (FC)	PAM treatment	5 min	24h	41,0%
	MDA-MB-468		PAM treatment	5 min	24h	46,0%
Merrill et al (2019)	MUM51	Annexin V/PI (FC)	Paclixatel	110 nM	72h	50,0%
	BT20			159 nM		
	BT549			110 nM		
	CAL148			4 nM		

CAL51		310 nM
DU4475		19 nM
HCC1806		77 nM
HCC1937		130 nM
HCC38		1700 nM
HCC70		3 nM
Hs578T		150 nM
MDA-MB-157		90 nM
MDA-MB-231		200 nM
MDA-MB-436		110 nM
MDA-MB-453		2 nM
MDA-MB-468		89 nM
MFM223		4 nM
SUM102		9 nM
SUM149		13 nM
SUM159		2 nM
SUM185		10 nM
SUM52		3 nM
MUM51	Docetaxel	2 nM
BT20		2 nM
BT549		1 nM
CAL148		2 nM
CAL51		4 nM
DU4475		5 nM
HCC1806		4 nM
HCC1937		1 nM
HCC38		1 nM
HCC70		1 nM

	Hs578T			1 nM		
	MDA-MB-157			1 nM		
	MDA-MB-231			2 nM		
	MDA-MB-436			1 nM		
	MDA-MB-453			1 nM		
	MDA-MB-468			1 nM		
	MFM223			740 nM		
	SUM102			1 nM		
	SUM149			5 nM		
	SUM159			140 nM		
	SUM185			2 nM		
	SUM52			2 nM		
Koechli et al (1993)	BT-20	ATP assay	Paclitaxel	0.00163 PPCs	90 min	50,0%
			Doxorubicin	0.319 PPCs		
			Paclitaxel and Doxorubicin	0.2277 PPCs		
Liu et al (2017)	MDA-MB-231	TrypanBlue	CAP tretament	120 s	48h	> 50%
	MDA-MB-453		CAP tretament	120 s	48h	> 20%
McCloskey et al (1996)	MDA-MB-468	TrypanBlue	Paclitaxel	17 nM	3h	50,0%
			Paclitaxel	2.6 nM	24h	
			Paclitaxel	1.8 nM	120h	
Kibria et al (2014)	MDA-MB-231	WST-8 assay	Doxorubicin	25.72±20.27μg/mL	8h	50,0%

Table S2 – Qualitative detailed of SYRCLE tool questions regarding *in vivo* studies.

	QUESTIONS									
**STUDY**	**1**	**2**	**3**	**4**	**5**	**6**	**7**	**8**	**9**	**10**
Man et al (2002)	Unclear	Unclear	Unclear	Unclear	Unclear	No	Unclear	Yes	Yes	Yes
Munõz et al (2019)	Unclear	Unclear	Unclear	Unclear	Unclear	Unclear	Unclear	Yes	Yes	Yes
Shaked et al (2016)	Unclear	Unclear	Unclear	Unclear	Unclear	Unclear	Unclear	Yes	Yes	Yes
Xiang et al (2018)	Unclear	Unclear	Unclear	Unclear	Unclear	Unclear	Unclear	Yes	Yes	Yes
Zhou et al (2020)	Unclear	Unclear	Unclear	Unclear	Unclear	Unclear	Unclear	Yes	Yes	Yes

Table S3 – Quantitative detailed of ToxRTool tool questions regarding *in vitro* studies.

	QUESTIONS																	
STUDY	**1**	**2**	**3**	**4**	**5**	**6**	**7**	**8**	**9**	**10**	**11**	**12**	**13**	**14**	**15**	**16**	**17**	**18**
Adachi et al (2018)	1	0	1	0	1	1	1	0	1	0	1	1	1	1	0	1	1	1
Almeida-Ferreira et al (2022)	1	0	1	1	1	1	1	0	1	0	1	1	1	1	1	1	1	1
Arun et al (2016)	1	0	1	1	1	1	1	0	1	0	1	1	1	1	0	1	1	1
Bernhardt et al (1992)	1	0	1	1	1	1	1	0	1	0	1	1	0	0	0	0	0	0
Blois et al (2011)	1	0	1	1	1	1	0	0	1	0	1	1	1	0	1	0	1	1

Chen et al (2018)<br>*Micro-sized cold atmospheric plasma source for brain and breast cancer treatment*	1	0	1	1	1	1	1	0	1	0	1	1	1	1	0	1	1	1
Chen et al (2017)<br>*Cold atmospheric plasma discharged in water and its potential use in cancer therapy*	1	0	1	1	1	1	1	0	1	0	1	1	0	1	0	0	1	0
Chen et al (2017)<br>*In vitro Demonstration of Cancer Inhibiting Properties from Stratified Self-Organized Plasma-Liquid Interface*	1	0	1	1	1	1	1	1	1	0	1	1	1	1	0	1	1	1
Cheng et al (2021)	1	0	1	1	1	1	1	0	1	0	1	1	1	1	0	1	1	1
Chuang et al (2012)	1	0	1	0	1	1	1	0	1	0	1	1	1	1	0	1	1	1
Di et al (2009)	1	0	1	1	1	1	1	0	0	0	1	1	1	0	1	1	1	1
Frankfurt & Krishan (2003)	1	0	1	0	1	1	1	1	0	0	1	1	1	0	1	0	1	1
Halfter et al (2016)	1	0	0	0	1	1	1	0	0	1	1	1	1	1	1	1	1	1
Hassan et al (2017)	1	0	1	0	1	0	0	0	1	0	1	1	1	0	0	1	1	1
Hernández-Vargas et al (2007)	1	0	1	1	1	1	1	0	1	0	1	1	1	1	0	0	1	1
Izbicka et al (2005)	1	0	1	1	1	1	1	0	1	0	1	1	0	1	0	1	1	1
Jezeh et al (2020)	1	0	1	1	1	1	1	1	1	0	1	1	1	1	1	1	1	1
Keung et al (2020)	1	0	1	1	1	1	1	0	1	0	1	1	1	1	0	1	1	1
Kibria et al (2014)	1	0	1	0	1	1	1	0	0	0	1	1	1	1	1	1	1	1
Kim et al (2003)	1	0	1	0	1	1	1	0	0	0	1	1	1	1	0	1	1	1
Koechli et al (1993)	1	0	1	0	1	1	1	0	1	0	1	1	1	1	0	1	1	1
Koechli et al (1994)	1	0	1	0	1	1	1	0	1	1	1	1	1	1	0	1	1	1

Konecny et al (2001)	1	0	1	0	1	1	0	0	1	0	1	1	1	1	0	1	1	1
Lafontaine et al (2020)	1	0	1	1	1	1	1	1	1	0	1	1	0	1	0	1	1	1
Liu et al (2017)	1	0	1	1	1	1	1	1	1	0	1	1	1	1	0	1	1	1
Ly et al (2020)	1	0	1	1	1	1	1	1	1	0	1	1	1	1	0	1	1	1
Ma et al (2017)<br>*Study on inhibitory effect of paclitaxel on MEK and ERK protein overexpression and activation in different breast cancer cell lines*	1	0	1	0	1	1	1	0	0	0	1	1	0	1	0	1	1	1
Ma et al (2020)<br>*Non-thermal plasma induces apoptosis accompanied by protective autophagy via activating JNK/Sestrin2 pathway*	1	0	1	1	1	1	1	0	1	0	1	1	1	1	0	1	1	1
McCloskey et al (1996)	1	0	1	1	1	1	1	0	0	0	1	1	1	1	0	0	1	1
Merrill et al (2019)	1	0	1	1	1	0	1	0	1	0	1	1	1	1	0	1	1	1
Mihai et al (2022)	1	0	1	1	1	1	1	1	1	0	1	1	1	1	0	1	1	1
Morse et al (2005)	1	0	1	0	1	1	1	0	1	0	1	1	1	1	1	1	1	1
Moschetta-Pinheiro et al (2022)	1	0	1	1	1	1	1	0	1	0	1	1	1	1	0	1	1	1
Munõz et al (2019)	1	0	1	1	1	0	1	0	1	0	1	1	1	1	0	1	1	1
Ninomiya et al (2013)	1	0	1	1	1	1	1	0	1	0	1	1	1	1	1	0	1	1
Norris et al (2013)	1	0	1	1	1	1	0	0	1	0	1	1	0	1	0	0	1	1
Oncul et al (2017)	1	0	1	0	1	1	1	0	1	0	1	1	1	1	1	1	1	1
Park et al (2015)	1	0	1	1	1	1	1	0	1	0	1	1	1	1	0	1	1	1
Parrella et al (2014)	1	0	1	0	1	1	1	0	0	0	1	1	0	1	0	0	1	1

Pilco-Ferreto & Calaf (2016)	1	0	1	1	1	1	1	0	1	0	1	1	1	1	0	1	1	1
Risinger et al (2015)	1	0	1	1	1	1	1	0	0	0	1	1	0	1	0	1	1	1
Sauter et al (1986)	1	0	1	0	1	1	1	0	0	0	1	1	0	0	0	0	0	0
Stope et al (2020)	1	0	1	0	1	1	1	1	1	0	1	1	1	1	0	1	1	1
Subramanian et al (2020)	1	0	1	1	1	0	1	1	1	0	1	1	1	1	0	1	1	1
Taherian et al (2012)	1	0	1	0	1	1	1	0	0	0	1	1	0	1	1	1	1	1
Tassone et al (2003)	1	0	1	0	1	1	1	0	1	0	1	1	1	1	0	1	1	1
Terefinko et al (2021)	1	0	1	1	1	1	1	1	1	0	1	1	1	1	0	1	1	1
Wali et al (2017)	1	0	0	0	1	0	0	0	1	0	1	1	0	0	0	0	0	1
Wang et al (2013)	1	0	1	1	1	1	1	1	1	0	1	1	1	1	0	1	1	1
Wang et al (2021)	1	0	1	1	1	1	1	1	1	0	1	1	1	1	0	1	1	1
Wesierska-Gadek et al (2015)	1	0	1	1	1	1	1	0	1	0	1	1	1	1	0	1	1	1
Xiang et al (2018)	1	0	1	1	1	1	1	1	1	0	1	1	1	1	0	1	1	1
Yan et al (2015)	1	0	1	1	1	1	1	1	1	0	1	1	1	1	0	1	1	1
Yan et al (2017)	1	0	1	1	1	1	1	0	1	0	1	1	1	1	0	1	1	1
Yaourtis et al (2023)	1	0	0	1	1	0	0	0	1	0	1	1	1	1	1	1	1	1
Zasadil et al (2014)	1	0	0	1	1	1	1	0	1	0	1	1	1	1	0	1	1	1
